# Effect of Thermal Stress on Morphology in High-Performance Organic Photovoltaic Blends

**DOI:** 10.1021/jacsau.4c00631

**Published:** 2024-10-10

**Authors:** Haoyu Zhao, Nathaniel Prine, Soumya Kundu, Guorong Ma, Xiaodan Gu

**Affiliations:** School of Polymer Science and Engineering, Center for Optoelectronic Materials and Devices, The University of Southern Mississippi, Hattiesburg, Mississippi 39406, United States

**Keywords:** bulk heterojunction, thermal stability, isothermal crystallization, morphological instability

## Abstract

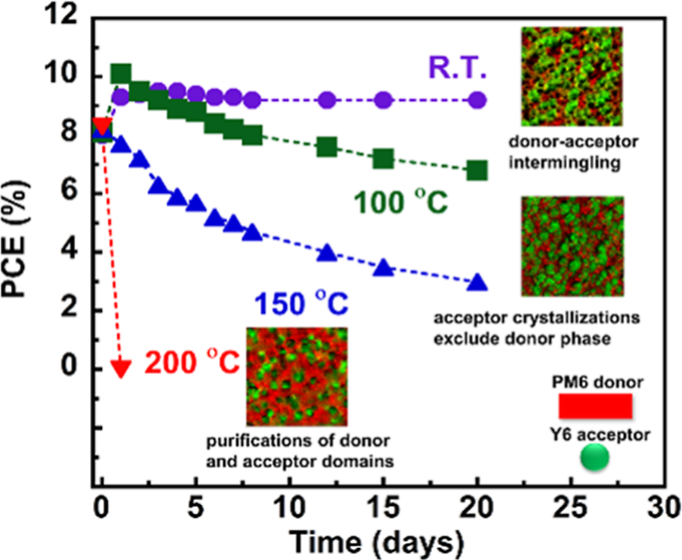

Thermal stress is a critical factor causing long-term instability in bulk heterojunction (BHJ) layers of organic photovoltaic (OPV) devices. This study provides direct insights into the thermal properties of Y6, PM6, and their binary blends by employing fast differential scanning calorimetry (flash DSC) to analyze their chain dynamics. The glass transition temperatures (*T*_g_) of Y6 and PM6 were measured, with Y6 exhibiting a *T*_g_ of 175.2 °C and PM6 showing two *T*_g_s at 39.7 and 107.6 °C. Our findings indicate that average OPVs’ operational temperatures are lower than the blend’s primary *T*_g_ of 138.2 °C. Thus, the mobility of PM6 and Y6 is not the critical factor that results in drastic drifts in the device morphology. Instead, we discovered that the crystallization of small molecules Y6 in the BHJ film at elevated operation temperatures *significantly contributes* to the morphological instability of the BHJ layer, based on a flash DSC isotherm crystallization study. The crystallization of the acceptor leads to severe phase separation between donors and acceptors and results in device failure. The acceptor Y6’s crystallization rate also increased when blended with donor PM6, compared to that of pure Y6 molecules. Furthermore, AFM–IR analysis of the morphology of the BHJ layer after high thermal stress of 200 °C revealed an apparent demixing of donor PM6 and acceptor Y6, revealing Y6 globules about 200 nm in diameter, with PM6 domains surrounding the Y6 regions. This crystallization-induced morphology change was later confirmed to correlate well with the device performance drop. This study offers valuable insights into the origin of BHJ layer instability in OPV devices containing nonfullerene small molecule acceptors and polymer donors. Additionally, it emphasizes the importance of addressing thermal stress to enhance the performance and durability of such devices and informs strategies for developing more stable organic solar cells.

## Introduction

Organic photovoltaics (OPVs) are a promising technique for providing renewable energy. Over the past two decades, there have been significant improvements in the power conversion efficiency (PCE) of these solar cells. However, most reported OPV devices are not stable for long-term operation. The generation of exciton and free charge carriers depends on the carefully tuned bulk heterojunction (BHJ) morphology.^1−4^ Exposure to oxygen, moisture, light, and heat causes the semistable BHJ layer to degrade chemically, mechanically, or morphologically. Previous efforts have mitigated extrinsic factors such as moisture and oxygen by sealing OPVs with flexible barriers^5−7^ or glass encapsulation^8−11^ to reduce photooxidation.^12^ The intrinsic factor for device stability is the chemical stability of the polymer, as well as the BHJ morphological stability.^13−19^ When the device operates at temperatures above the *T*_g_ of both the donors and acceptors, materials in the photoactive BHJ layer undergo a glass transition and become mobile. Thus, the *T*_g_s for donors and acceptors should be designed at higher temperature (>100 °C) to resist segmental movement when the devices operate at elevated temperatures, where extremely hot environments and/or lack of ventilation/insufficient airflow can significantly increase the surface temperature of devices; for example, amorphous silicon solar cells have been shown to reach junction temperatures beyond 110 °C when exposed to sunlight.^20^ If the photovoltaic materials do not have a high *T*_g_, they may begin to self-aggregate and crystallize during device operation.^21,22^ Additionally, donors and acceptors could phase separate, moving away from the nonequilibrium metastable morphology upon film deposition, leading to a rapid decline in OPV performance.^13,22,23^ Therefore, it is beneficial to select OPV donors and acceptors that possess high *T*_g_, relative to their expected device operating temperature.

Semicrystalline polymer donors PBDB-T-2F (PM6) and small molecule nonfullerene acceptors (NFAs) Y6 have recently achieved PCEs above 18%^24^ due to their complementary energy levels and optimized device morphology.^24^ Despite the impressive PCE for newly fabricated devices, they generally lack stability for extended operation. OPV device performance decays over time due to the fact that the BHJ layer is generally not in a thermodynamic equilibrium state. Any segmental mobility would result in a drift in the BHJ morphology. While PM6/Y6-based OSCs continue to advance their PCE, there has been limited study of the degradation mechanism. Hence, we chose PM6 and Y6 as our model system here, and our study aims to address this gap by providing a comprehensive investigation of the morphology evolution in PM6/Y6-based OPVs under thermal stress.^22^

Several studies have reported on the mechanism of instability for BHJs involving NFAs. He and co-workers discovered that the thermal stability of the BHJ of PM6 and ITIC-4F NFA could be dramatically enhanced through the strategic addition of (1,8-diiodooctane) DIO additives.^25^ The addition of DIO increased the crystallinity and coherence length of the NFA without affecting the morphology of the PM6 donor.^25^ This finding suggests that the mobility of small molecule NFAs directly impacts device stability. Zhang et al. blended an asymmetric NFA, IDST-4F, with PM6 to achieve enhanced thermal stability.^26^ After thermally annealing the blend at 150 °C for 20 h, the OPV device retained 80% of its initial PCE.^26^ Similarly, addition of a third component, fullerene derivatives, has been reported to improve the stability of PM6/Y6-based OPVs.^27−30^ Lastly, Ade and co-workers reported that the Flory–Huggins interaction parameter, χ, which describes the free energy of mixing (Δ*X*_G_) for two polymers, is a predictor of device stability.^31,32^ This interaction parameter is temperature-dependent, and spinodal decomposition of the BHJ is thermally accelerated. Ade’s group has adopted this framework and applied it to BHJ systems. The Flory–Huggins theory is highly useful for describing the thermal equilibrium phase behavior, although BHJs can be trapped in a nonequilibrium state where the theory cannot fully capture the dynamics.

In this study, we used PM6 and Y6 as a model system to study the device morphology and performance at a wide range of operating temperatures to understand the underlying mechanism for device performance loss. First, we measured the thermal transitions of PM6, Y6, and their BHJ blends. Using advanced thermal characterization by flash differential scanning calorimetry (DSC), we quantified the *T*_g_s as well as the crystallization behavior of Y6 molecules and its BHJ blends. Second, wide-angle X-ray scattering (WAXS) was used to measure the crystalline domain growth of the donor/acceptor at different temperatures. Furthermore, we utilized atomic force microscopy paired with infrared spectroscopy (AFM–IR) to directly measure and understand the domain size and purity of binary blends annealed at various temperatures. Finally, we constructed solar cell devices and compared the morphology with device performance loss. We observed that the crystallization of the acceptor small molecules Y6 in the blend film significantly contributes to the BHJ layer’s morphological instability, resulting in severe phase separation between donors and acceptors. By gaining a deeper understanding of the morphology evolution in PM6/Y6-based OPVs at high operating temperatures, this study could contribute to the development of more efficient, stable, and commercially viable OPVs in the future.

## Results and Discussion

### Thermal Properties and Crystallization Behaviors of the Donor, Acceptor, and Blend

**Figure 1:**
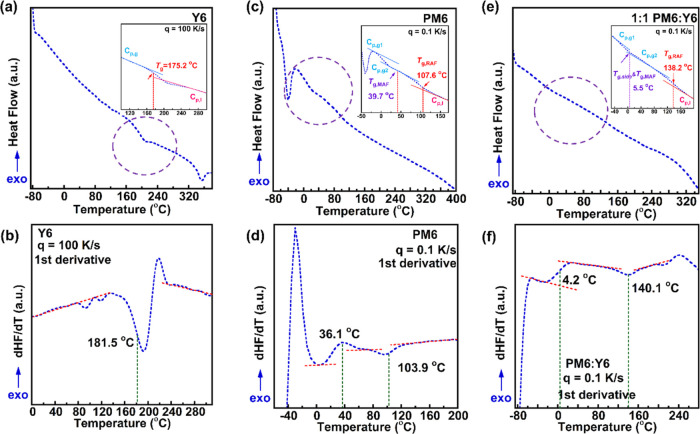
Thermal signatures of the Y6 acceptor, PM6 donor, and 1:1 blend ratio BHJ film: (a) flash DSC thermal analysis for probing the *T*_g_ for the Y6 acceptor, (b) corresponding 1st derivative of Y6 glass transition regions, (c) flash DSC thermal analysis for probing the *T*_g_ for the PM6 donor, (d) corresponding 1st derivative of PM6 glass transition regions, (e) flash DSC thermal analysis for probing the *T*_g_ for the 1:1 BHJ blend film, and (f) corresponding 1st derivative of blend glass transition regions, where inset figures indicate the step change of *T*_g_.

Thermal stress is a major factor contributing to the long-term instability of the BHJ layer.^12^ Consequently, it is crucial to investigate the thermal signatures of the donor, acceptor, and blend films to understand their responses to temperature fluctuations under various operational conditions. We begin our characterization of the thermal properties by measuring the *T*_g_ of neat Y6 (Figure 1a), neat PM6 (Figure 1c), and a 1:1 (w/w) BHJ blend (Figure 1e) using flash DSC at cooling rates ranging from 0.1 to 4000 K/s. We could not measure the *T*_g_ of PM6 or Y6 using conventional DSC due to the weak signal coming from the small conformational changes during thermal transitions caused by their rigid backbones and complex molecular structures.^33^ However, flash DSC allows samples to cool rapidly, inhibiting crystallization during the cooling step and amplifying glass transition signals for improved signal detection.^34,35^ Therefore, the *T*_g_ of Y6 at 175.2 °C was revealed using a cooling rate (*q*) of 100 K/s for the well represented one (Figure 1a inset). The slowest cooling rate of 0.1 K/s for *T*_g_ of Y6 can be found in Figure S1. Additionally, Y6 showed a clear melting peak at around 340 °C but no alkyl side chains relaxations at low temperature ranges (e.g., between ∼−50 and 0 °C). The reason that lacking side chain *T*_g_ for Y6 could be attributed to the strong backbone π–π interactions that thermal behaviors were dominated by backbone relaxations resulting in masked side chain relaxations. In addition, the highly ordered side chain packing and/or the relatively lower volume fractions of side chains may also impact the appearance of distinct side chain *T*_g_. The PM6 donor displayed quite different thermal properties, as shown in Figure 1c. The side chain *T*_g_ stood out at temperatures ranging from −60 to −30 °C, indicating a highly mobile side chain at room temperature. Additionally, we observed two additional *T*_g_s for neat PM6, and we assigned the lower *T*_g_ (halfway between the first specific heat capacity glassy line *C*_p,g1_ and the specific heat capacity liquid line *C*_p,l_) at 39.7 °C to the mobile amorphous fractions (MAFs) of the backbone and the higher *T*_g_ (halfway between the second specific heat capacity glassy line *C*_p,g2_ and the specific heat capacity liquid line *C*_p,l_) at 107.6 °C to the *T*_g_ of rigid amorphous fractions (RAFs) of the backbone,^36^ where the existence of two *T*_g_s was also confirmed by the DMA test (as shown in Figure S2). Finally, the lack of melting peaks up to 400 °C indicates that PM6 does not have long-range ordered structures and is classified as a 2D amorphous structure.^37^ Once blended, the 1:1 mixed BHJ sample showed interesting thermal transition features inherent from both donors and acceptors, as shown in Figure 1e. The lower temperature thermal transition of 5.5 °C is the combined relaxations from side chain and the MAFs of the backbone, and the higher *T*_g_ of 138.2 °C is the primary one, which lies in between PM6 and Y6, as predicted by the Fox equation.^38^ We are more interested in the correlations between operational stability and *T*_g,RAF_ since the thermodynamics state of the RAF is tightly related to the degree of crystallinity^36^ and morphology.^39^ The first derivative plots of heat flow for Y6, PM6, and the 1:1 blend are illustrated in Figure 1b,d,f, respectively. The *T*_g_ value can also be determined from the halfway value between steps in the first derivative plots. For neat Y6, the large enthalpy overshoot occurred after the slower cooling rate so that abrupt peaks were shown during the enthalpy overshoot range (131–232 °C), resulting in a slightly off halfway value of 181.5 °C in Figure 1b. The observed *T*_g_ value of 138.2 °C for the BHJ is higher than typical operational temperatures for OPVs (70–110 °C). As a result, this implies that donor–acceptor phase separation caused by coorporative mobile chain movement is not the primary factor contributing to device instability. We later performed a detailed characterization to confirm this.

**Figure 2:**
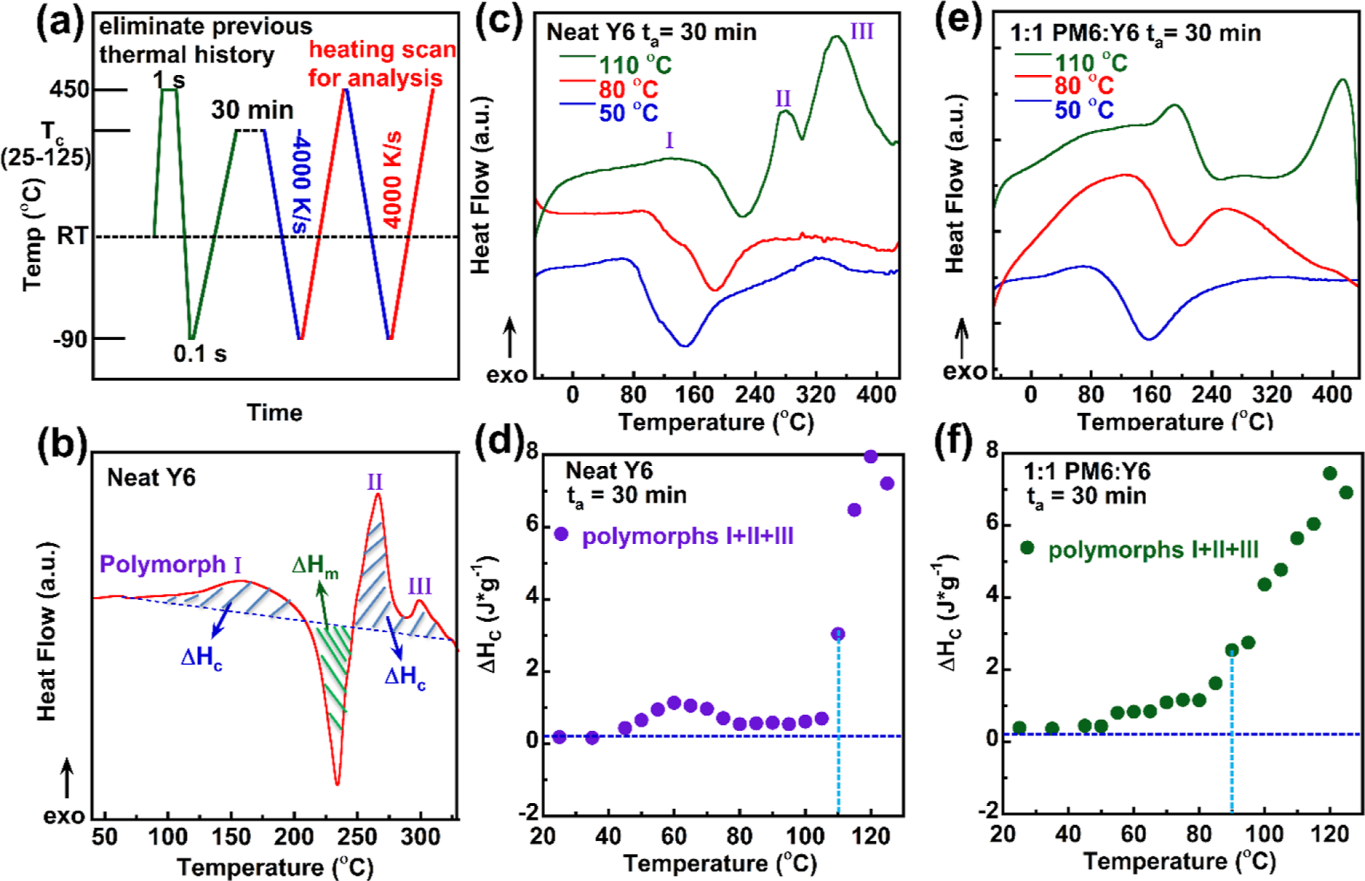
Using flash DSC to measure the crystallization behavior of the Y6 acceptor and BHJ film. (a) Temperature protocol for flash DSC isothermal crystallization studies, (b) schematic heating plots for enthalpy changes upon crystallization, (c) representative Y6 heating scans after 30 min isothermal crystallization at 50, 80, and 110 °C scanning temperatures, (d) combined enthalpy changes (Δ*H*_C_) upon crystallization against annealing temperature for neat Y6, (e) representative 1:1 blend film heating scans after 30 min isothermal crystallization, and (f) combined enthalpy changes (Δ*H*_C_) upon crystallization against annealing temperature for the 1:1 blend, where polymorphs are named in order as the crystallization peak temperatures increase from room temperature to the end of the heating scan.

Next, we examined the crystallization behaviors of the neat acceptor Y6, which has been previously reported as a major factor affecting the performance of OSC devices.^40^ Contrary to conventional polymers, which can only crystallize above their *T*_g_, small molecule acceptors (e.g., Y6 or ITIC) can undergo a glass–crystal transition. This crystallization process is driven by the diffusion of small molecules, and the crystallization onset temperature may be significantly lower than its *T*_g_.^41^ We conducted isothermal crystallization studies using flash DSC, leveraging its unique cooling capability. The temperature protocol employed, illustrated in Figure 2a, utilized a cooling and heating rate of 4000 K/s. This rapid cooling later ensured that the sample crystallization could be inhibited so that it would stay amorphous before isothermal annealing. The isothermal crystallization temperatures (*T*_c_) in this studied ranged from 25 to 125 °C, with a fixed isothermal crystallization time (*t*_a_: annealing time) of 30 min. Figure 2b is a representative crystallization behavior for the Y6 molecule upon a heating scan following an isothermal crystallization. The sample showed several polymorphs for its crystallites. The initial exothermic peak between 100 and 200 °C corresponds to polymorph I, while subsequent exothermic crystallization peaks near 260 and 300 °C are for polymorphs II and III, respectively. Figure 2c depicts flash DSC results for neat Y6 annealed at 50, 80, and 110 °C, with the appearance of multiple polymorphs. By integrating the crystallization peaks (as shown in Figure 2b), we can establish correlations between the enthalpy changes upon crystallization (Δ*H*_c_) and examine crystal growth as a function of crystallization temperatures (Figure 2d). As the annealing temperature increases, all polymorphs (I, II, and III) grow, with a major enhancement observed at 110 °C, suggesting that more Y6 crystals form at this temperature, as evidenced by the significantly higher Δ*H*_c_. Conversely, Figure 2e,f displays crystallization studies for the 1:1 PM6/Y6 BHJ film across the same temperature range, leading to a similar conclusion of increased crystallinity at higher annealing temperatures. However, isothermal crystallization of Y6 accelerated in the blend film, as indicated by the sharply enhanced Δ*H*_c_ at 90 °C relative to neat Y6, predominantly due to the increase of polymorph I. (The detailed polymorphs against annealing temperature data can be found in Figure S3.) Based on the flash DSC result, neat Y6 clearly demonstrates its ability to crystallize well below its *T*_g_ due to its ability to diffuse. Additionally, more crystals form around 110 °C for neat Y6 molecules, and this enhanced isothermal crystallization shifts to a lower temperature of 100 °C for the BHJ blend film. The impacts of 100 °C isothermal crystallization upon device stability can be verified by annealing the active layer for varied time frames. The device performance (PCE) for the 100 °C 1 h annealed active layer was similar to that for the room temperature as-cast device, but the PCE significantly declined at longer annealing time due to the rapid growth of crystals, for which the detailed performance data can be found in Figure S4.

**Figure 3:**
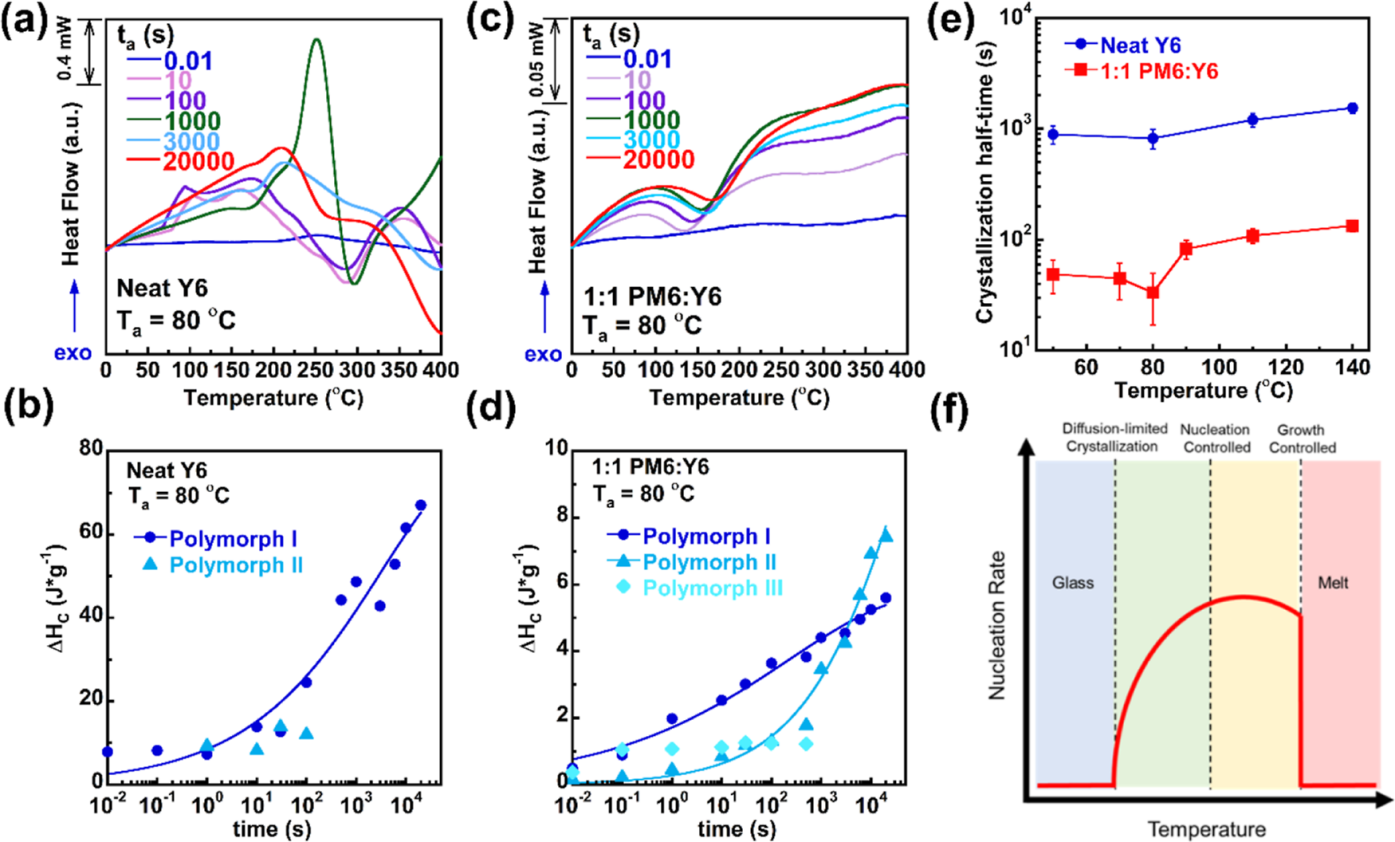
Representative isothermal crystallization kinetics investigations for (a) neat Y6 at 80 °C and (c) 1:1 PM6/Y6 at 80 °C; enthalpy changes upon crystallization against isothermal annealing times for (b) neat Y6 and (d) 1:1 blend; (e) isothermal crystallization half time vs annealing temperatures for neat Y6 and 1:1 blend; and (f) schematic plots of temperature influence upon crystallization.

To further investigate the observed differences in isothermal crystallization behaviors between neat Y6 and the blend film, a detailed crystallization kinetics investigation was then performed. Here, we will discuss representative crystallization kinetics data at 80 °C with isothermal annealing times ranging from 0.01 to 20,000 s, as shown in Figure 3. Additional crystallization kinetic studies at different annealing temperatures can be found in Figures S5–S7. For neat Y6, the crystallization peak area increased rapidly with the longer isothermal annealing times, indicating that small crystalline domain formed and grew into larger crystals within 1000 s. The area under the enthalpic peak was integrated to analyze the change in Δ*H*_c_ over the annealing time, as shown in Figure 3b. The crystallization kinetics was analyzed by applying the Avrami equation^42^ as follows
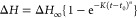
1where Δ*H*_∞_ is the final enthalpy changes upon primary crystallization, *t*_0_ is the induction time, *K* is the crystallization rate constant, and *n* is the Avrami index. For neat Y6 isothermally crystallized at 50 and 80 °C, both polymorph I and polymorph II were formed in short time periods (0.01–0.1 s for 50 °C and 1–100 s for 80 °C). Nonetheless, after annealing time was prolonged, polymorph I crystal sizes were significantly increased and polymorph II was excluded for the temperatures ranging from 0 to 400 °C, as observed by much higher enthalpy changes upon crystallization (Δ*H*_c_) that were proportional to the weights of crystals. For higher annealing temperatures (110 and 140 °C), although polymorph II was discernible until an annealing time of 10,000s, the larger gap between enthalpy changes between polymorph I and polymorph II at the same temperature still indicated that the isothermal crystallization was dominated by Y6 polymorph I. Therefore, Avrami fittings were adopted on the dominating polymorph I with the most enthalpy changes upon crystallization, and the Avrami fitting parameter in Table S1 illustrates that the crystallization rate constant was similar for all selected isothermal crystallization temperatures within errors, where the variations in Avrami index (*n*) indicate that the growth geometry changed at higher annealing temperatures.

After the initial study of neat Y6 molecules, we conducted the same annealing procedure for the PM6/Y6 1:1 blend ratio BHJ film (Figure 3c). The blend films underwent crystallization, melting, and then a recrystallization process after the initial 0.01 s of annealing, resulting in both polymorph I and polymorph II being observed in Figure 3d. Polymorph I in the blend film still dominated crystal growth for shorter annealing times, but secondary crystallization of polymorph II increased rapidly with longer annealing times. Eventually, a high degree of crystallinity formed in the blend film over the course of the experiment, evidenced by the high enthalpy peak from the combination of both polymorphs. For polymorph I, the crystallization half-time can be calculated using the following equations
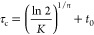
2where τ_c_ is the crystallization half time and *K* and *n* are the overall crystallization rate constant and Avrami index (the fitting parameters are listed in Table S2), respectively. The crystallization half-time for neat Y6 and the PM6/Y6 film was plotted as a function of temperatures, as shown in Figure 3e. Y6 showed a higher crystallization half time than the blend film at all annealing temperatures, indicating a slower crystallization rate than that of BHJ blends by almost a factor of 10. The faster crystallization rate observed in the blend could be attributed to the enhanced mobility from lower *T*_g_ of donor contribution, where donor/acceptor interactions may provide an ideal nucleation site for the crystal to rapidly grow. The schematic representation of the crystallization rate vs annealing temperature plot (Figure 3f) summarized our observation that the nucleation rate increased steadily during the diffusion-limited regime, reaches a maximum rate when transitioning into the nucleation-controlled regime, and finally drops as the sample enters the melting regime. Therefore, the origin of the unstable device performance at elevated temperature could be correlated with the rapid crystallization of NFA Y6 in the BHJ.

### Temperature-Dependent Morphological Investigations of the Donor, Acceptor, and Blend Active Layer

**Figure 4:**
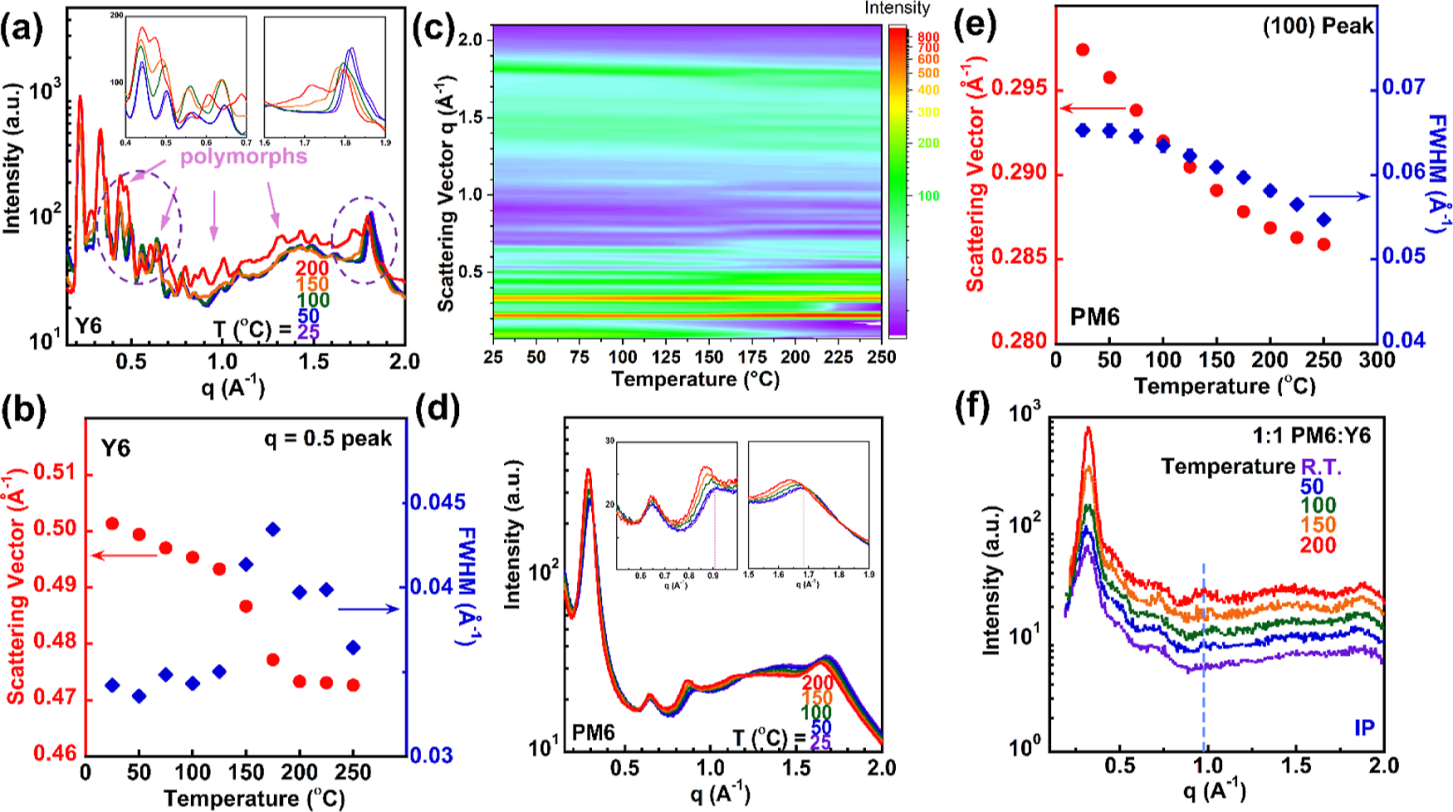
(a) WAXS experiments for bulk Y6, (b) fitting results for the *q* = 0.5 peak of Y6, (c) corresponding intensity map of Y6, (d) WAXS experiments for bulk PM6, (e) fitting results for the (100) peak of PM6, and (f) GIWAXS experiments for 1:1 PM6/Y6 after specific ex situ thermal treatment.

Temperature-dependent WAXS was used to further examine the crystallization behaviors of NFA Y6 and BHJ. The in situ WAXS result for Y6, shown in Figure 4a, confirmed that multiple polymorphs emerged, evidenced by the appearance of several new scattering peaks. The scattering vector ranging from 0.4 to 0.7 Å^–1^ was magnified to further examine the growth of these peaks, as shown in the inset in Figure 4a. Comparing room temperature samples with those heated to 150 °C, a newly formed scattering peak at *q* = 0.55 Å^–1^ was observed (as shown in Figure S8a), correlating to polymorph I identified in flash DSC measurements. After heated to 200 °C, Y6 recrystallized into polymorph II (Figure 2b), with new peaks forming at *q* = 0.69, 0.89, 1.0, 1.3, 1.4, and 1.7 Å^–1^, as indicated by arrows in Figure S8b. Additionally, around *q* = 1.82 Å^–1^, the π–π packing peak was observable at all temperatures but slightly shifted to a lower *q* due to lattice expansion with increasing temperatures. Moreover, we tracked the peak shift and full width at half-maximum (FWHM) to probe the temperature influences for fitting the scattering peak initially at *q* = 0.5 Å^–1^. Figure 4b clearly shows the peak position change and the expansion of FWHM from 100 to 200 °C, which confirmed the influences of crystallizations induced by a higher annealing temperature. Figure 4c presents the corresponding intensity map for the Y6 WAXS results, where *q* = 0.44 Å^–1^ scattering peaks intensified as the temperatures increased. In addition to that, the scattering peak around *q* = 0.64 Å^–1^ separated into two peaks at *q* = 0.61 Å^–1^ and *q* = 0.68 Å^–1^ as Y6 was heated beyond 150 °C, as indicated by the arrows in Figure S8c. On the other hand, Figure 4d shows WAXS results for neat PM6, exhibiting typical semicrystalline polymer scattering peaks, including (100), (200), and (300) lamellar packing peaks and the (010) π–π packing peak. Elevated temperatures caused the π–π peak to shift to a lower *q* range due to thermal expansion, while the (300) peaks grew at higher temperatures, as depicted in the inset figures of Figure 4d. The thermal expansion at elevated temperatures also slightly influenced the scattering peak position and FWHM from the fitting results of the (100) peak shown in Figure 4e. Finally, the similar intensity map of PM6 (as shown in Figure S9) was established, and no polymorph was observed for the donor polymer.

Next, the thin film 1:1 PM6/Y6 BHJ film was investigated by grazing incidence WAXS (GIWAXS), as shown in Figure 4f. Preparing a BHJ for very thick bulk samples is technically challenging, so the GIWAXS technique was used to track the crystallization behavior. The in-plane 1-D plots displayed all films annealed at different temperatures for 24 h. The scattering peaks remain nearly identical before and after annealing below 100 °C. However, a weak and broad scattering peak at *q* = 0.97 Å^–1^ appeared, representing the newly formed polymorphs from Y6 at higher annealing temperatures above 150 °C, as indicated by the dashed line in Figure 4f (the fitting peak information can be found in Table S3). The corresponding 2D images of in situ WAXS and ex situ GIWAXS can be found in Figures S10, S11 and S12–S16, respectively.

**Figure 5:**
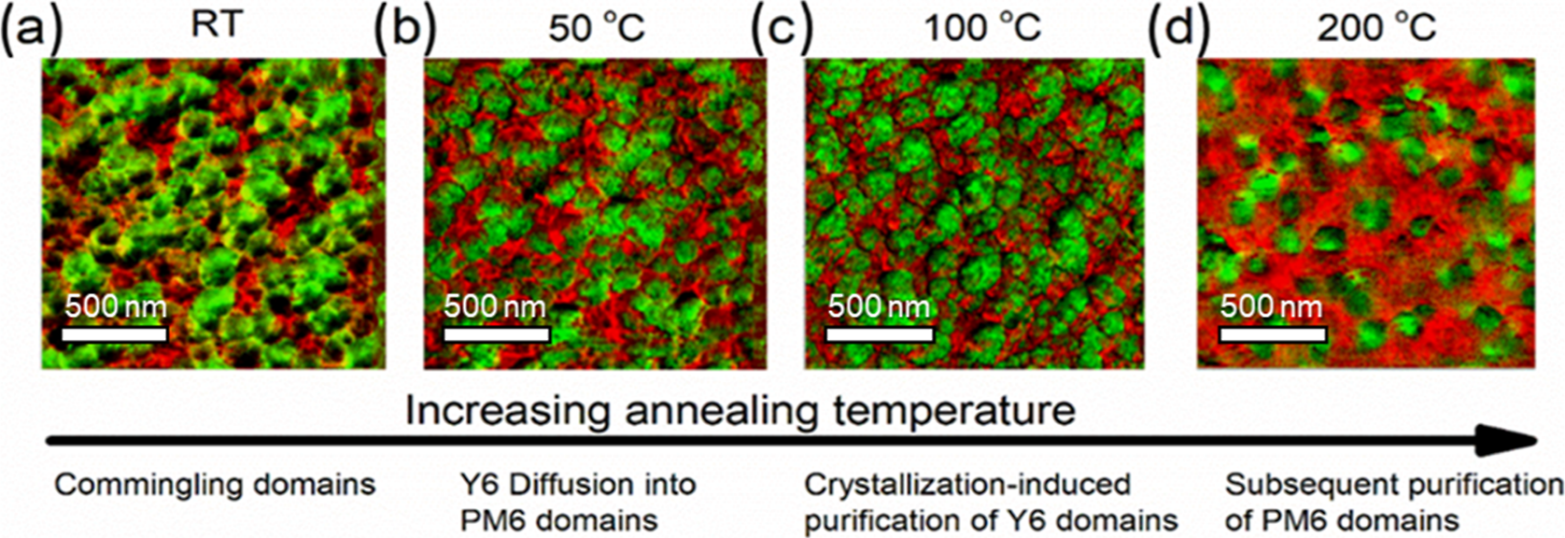
AFM–IR overlay image depicting the nanoscale morphology blends of PM6/Y6 (1:1) after annealing at (a) room temperature, (b) 50, (c) 100, and (d) 200 °C. PM6 domains are highlighted in red, and Y6 domains are highlighted in green.

AFM–IR was utilized to evaluate the morphology of PM6/Y6 blends at room temperature (as cast) and after annealing at 50, 100, and 200 °C for 8 h (Figure S17). Prior to AFM–IR measurements, we conducted bulk Fourier transform infrared spectroscopy (FTIR) to identify suitable wavenumbers to selectively excite either donors or acceptors by locating their distinct absorption peaks, as shown in Figure S18. The IR laser was first tuned to excite the PM6 phase at 1666 cm^–1^ and then to excite Y6 at 2217 cm^–1^. Two IR images collected at different wavenumbers were overlaid to visualize both PM6 and Y6 domains in a single image (Figure 5), where red regions represent PM6-rich domains and green regions represent Y6-rich domains. For the as-cast film, PM6 and Y6 exhibit intermingling domains with PM6 fibrils interpenetrating Y6 domains (Figure 5a). As the temperature increases further to 50 and 100 °C, Y6 domains grow and phase separate from PM6 (Figure 5b,c). At higher temperatures, the PM6 and Y6 domains gain further segmental mobility, disrupting the bicontinuous morphology, resulting in a disconnected, spherical domain of Y6 (Figure 5d).

**Figure 6:**
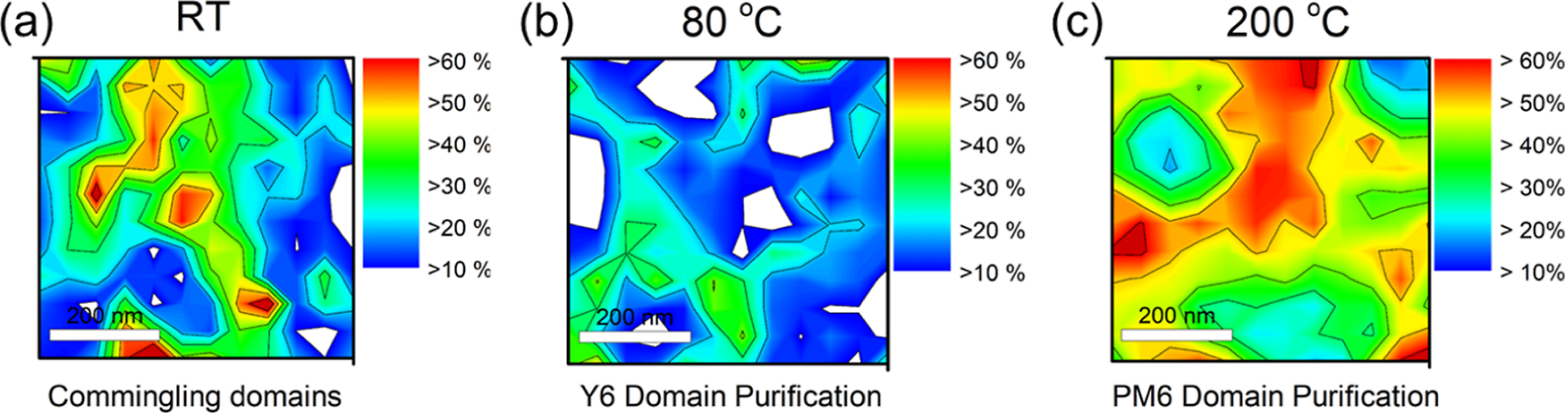
AFM–IR composition plots for the (a) PM6/Y6 blend film at room temperature, (b) PM6/Y6 blend annealed at 80 °C for 8 h, and (c) PM6/Y6 blend annealed at 200 °C for 8 h. The scale bar shows the percentage of PM6 in the mixed domain.

AFM–IR was used to collect a broadband IR spectrum with nanoscopic resolution from BHJ films to investigate their domain purity. The PM6 composition in each image was determined by measuring the AFM–IR response at each location, calculating the signal area ratio of the two materials, and comparing the result to the calibration curve (see Supporting Information Figure S19 and our previous work on this technique).^43^ This allowed us to ascertain the precise PM6 composition at the scan location. Based on the nanoscopic AFM–IR spectral signals, we calculated the localized percentage of PM6 composition for each sample (Figure 6). For the fresh PM6/Y6 blend, we observed domains with a PM6 composition greater than 60% (Figure 6a). After annealing at 80 °C, a reduction in PM6 composition was noted with small domains displaying less than 10% PM6 (Figure 6b). These smaller domains are primarily composed of Y6 and continue to grow at higher temperatures, confirming our previous observation that Y6 domain crystallizes. Lastly, after annealing at 200 °C, a dramatic increase in PM6 composition was noted in specific areas, with larger domains displaying more than 60% PM6 composition (Figure 6c). These figures show that domain purity increases with phase separation as the sample is heated to the crystallization temperature of the Y6 molecule. As the temperature increases to 200 °C, PM6 gains segmental mobility after reaching its *T*_g_, aiding in phase separation with Y6 domains. These observations of domain purification and morphological changes at different annealing temperatures have important implications for the performance and stability of organic solar cells. The purification of domains and the changes in PM6 and Y6 compositions could impact the efficiency of charge separation and transport within the active layer.

### Photovoltaic Characteristics of Fresh and Thermally Aged PM6/Y6 Solar Cells

**Figure 7:**
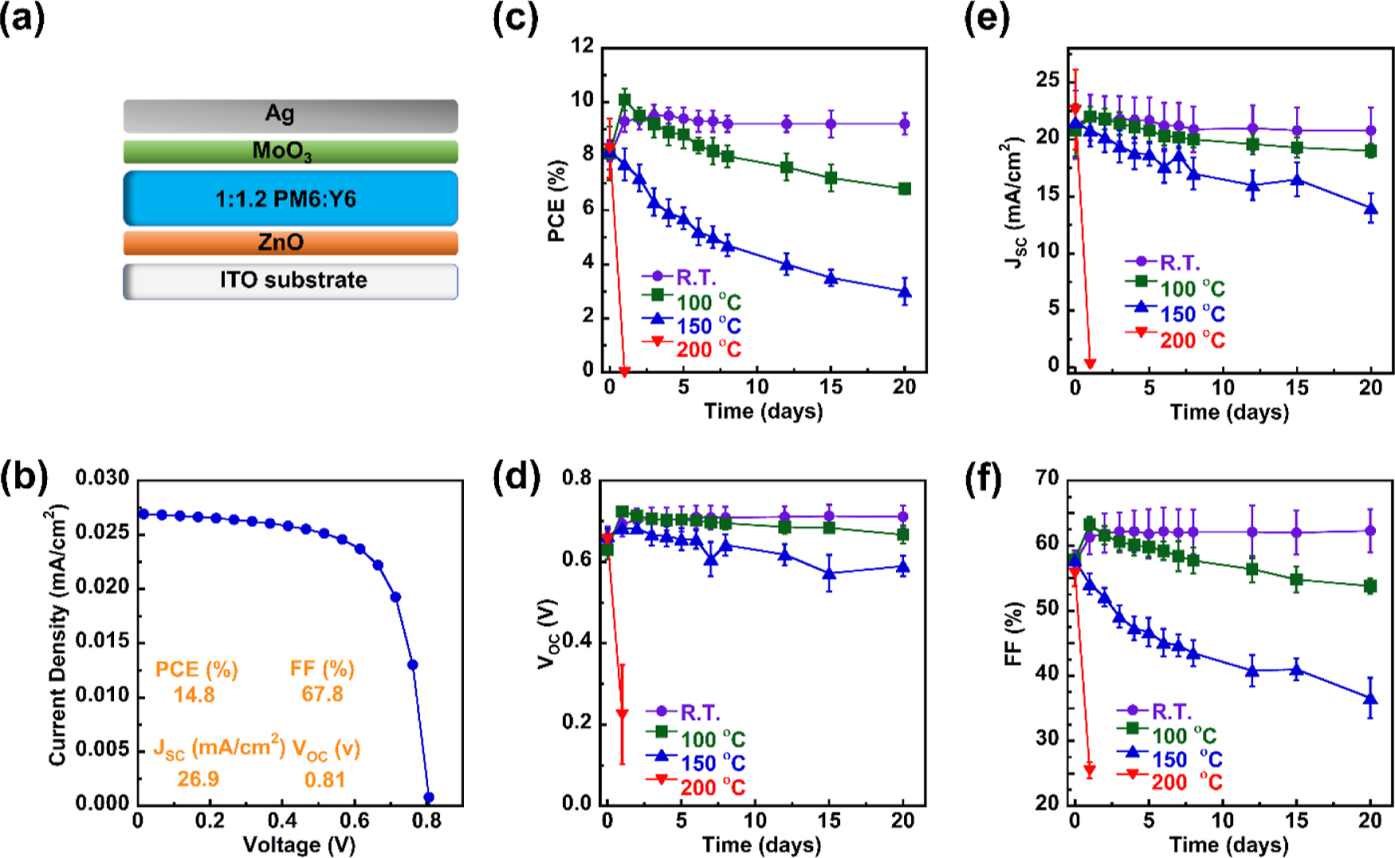
OSC device characterizations: (a) schematic representation of the inverted geometry of the solar cell device, (b) representative *J*–*V* curve of the PM6 and Y6 solar cell; and effects of thermal annealing on PM6/Y6 solar cell parameters annealed at different temperatures and times: (c) PCE, (d) *V*_OC_, (e) *J*_sc_, and (f) FF.

Lastly, we investigated the thermal stability of the PM6/Y6-based solar cell devices to correlate device properties with morphology characterization. We fabricated inverted devices with the structure of ITO/ZnO/PM6/Y6/MoO_3_/Ag (as shown in Figure 7a). The complete devices were annealed at different temperatures inside a nitrogen-filled glovebox. Initially, we optimized the device to reproduce the results reported in the literature based on the PM6/Y6 blend.^44−46^ Once we obtained reliable devices with a PCE over 12% (detailed parameters in Table S4), we proceeded to the thermal stress test. Thermal annealing/aging was performed at room temperature, 100, 150, and 200 °C in the glovebox for up to 20 days. In Figure 7b, we plotted the representative current density–voltage (*J*–*V*) characteristics of the PM6/Y6 devices without annealing, whereas other annealing conditions for *J*–*V* curves can be found in Figure S20. Furthermore, the averaged PCE, open-circuit voltage (*V*_OC_), short-circuit current density (*J*_SC_), and fill factor (FF) as a function of annealing time over 20 days were plotted (Figure 7c–f), respectively. These photovoltaic parameters are summarized in Table 1, and the rest of the data can be found in Table S5. Devices at room temperature showed an averaged PCE of 8.0 ± 0.5%, with a *J*_SC_ of 21.3 ± 3.0 mA/cm^2^, a *V*_OC_ of 0.652 ± 0.035 V, and an FF of 57.5 ± 1.7% initially, and no performance loss was observed after 20 days of aging time without thermal stress, where the averaged PCE of 9.2 ± 0.5% was retrieved for day 20th data. Upon thermal annealing at 100 °C, a monotonically downward trend of device performance was observed after 4 days due to reductions in all photovoltaic parameters, as shown in Table 1. The thermal degradation was more severe for the device annealed at 150 °C, where performance began to decline after day 1, leaving only 30% of the performance at the end of the 20 day stability test. At 200 °C, the effects were so profound that the PCE dropped sharply to 0.001 ± 0.0005% after 1 day of annealing, and no further stability tests were conducted since the devices could no longer function.

**Table 1:** Time-Dependent OSC Device Performance Measurements at Various Annealing Temperatures for Represented Annealing Days

no. of devices characterized	*T*_anneal_ (°C)	time (days)	PCE (%)	FF (%)	*J*_SC_ (mA/cm^2^)	*V*_OC_ (V)
11	R.T.	0	8.0 ± 0.5	57.5 ± 1.7	21.3 ± 3.0	0.652 ± 0.035
11	R.T.	7	9.3 ± 0.4	62.0 ± 3.6	21.2 ± 2.0	0.709 ± 0.028
9	R.T.	20	9.2 ± 0.4	62.3 ± 3.3	20.8 ± 2.0	0.711 ± 0.028
10	100	0	8.1 ± 1.0	57.9 ± 0.9	20.8 ± 2.3	0.630 ± 0.024
10	100	7	8.2 ± 0.5	58.4 ± 2.3	20.2 ± 0.8	0.698 ± 0.015
11	100	20	6.8 ± 0.2	53.8 ± 1.2	19.0 ± 0.6	0.667 ± 0.022
11	150	0	8.2 ± 0.4	57.7 ± 0.5	21.5 ± 1.0	0.664 ± 0.018
16	150	7	5.0 ± 0.4	44.7 ± 1.7	18.6 ± 1.5	0.607 ± 0.042
14	150	20	3.0 ± 0.5	36.6 ± 3.1	14.0 ± 1.3	0.590 ± 0.025
8	200	0	8.3 ± 1.1	55.8 ± 2.0	22.6 ± 3.5	0.656 ± 0.022
11	200	1	0.001 ± 0.0005	25.5 ± 1.2	0.3 ± 0.2	0.225 ± 0.122

To directly correlate device morphology with performance, we conducted optical microscopy and AFM–IR on the same OPV full devices after thermal stress and device characterization. Some devices showed drastic changes in film quality and morphology that could be observed by optical microscopy. In Figure S21, both room temperature and 100 °C aged samples had a smooth topography without any visible domain formations. However, a large increase in film inhomogeneity can be observed for the 200 °C annealed sample. Large crystallites appeared, and their concentration increased rapidly with an annealing time. After 8 h, the film surface was dominated by crystallites due to rapid crystallizations of Y6 acceptors, aligning with the study of isothermal crystallization behaviors examined by flash DSC.

Next, AFM–IR experiments revealed the temperature influence on the device stability. As shown in the corresponding AFM height images (Figure S22), film roughness increased from 1.3 nm (room temperature) to 1.5 nm (100 °C annealed 8 h) and eventually to over 12 nm (200 °C annealed 8 h), confirming the significantly enhanced roughness due to Y6 crystallite formation. The chemical compositions of these crystallites were investigated using AFM–IR nano spectra. In Figure S23, an IR laser was applied to scan the surface and showed that higher and stronger regions (location 1) displayed a Y6 peak at 2214 cm^–1^ (Figure S23d), while lower and weaker regions (location 2) showed flat signals, indicating that the crystallites were formed by Y6 aggregation. Finally, similar PM6/Y6 chemical compositions overlay images are presented in Figure S24 for the full solar cell devices, where red and green areas are assigned to PM6-rich and Y6-rich regions, respectively, on ITO glasses. The initial comingle domains gradually shifted to phase-separated PM6 domains and Y6 domain as the annealing time increased from 1 to 8 h at 100 °C. For the devices annealed at 200 °C for 1 and 8 h, samples displayed large domain sizes, which is the reason that contributes to device failure. Here, we can conclude that the crystallization of Y6 is correlated to the larger and purer domains, which will contribute to device performance loss. Once the *T*_g_ of the donor PM6 is crossed, both domains become mobile and large phase separation occurs, which results in failure of the device.

## Conclusions

Our work suggests that merely maintaining temperatures below the *T*_g_ is insufficient to ensure morphological stability for the polymer (PM6) and small molecule (Y6) BHJ device. It is vital to consider additional factors, such as the diffusion-limited crystallization rate, which substantially affects morphological transformations and domain purity change. Particularly, the crystallization of Y6 molecules is responsible for the morphology drift for the PM6/Y6 device and, hence, the loss of the device performance. Consequently, future research endeavors will concentrate on deciphering the relationship between crystallization rates and morphological alterations, extending the applicability of these insights to other materials, and devising innovative approaches to inhibit the acceptor crystallization at the device operational temperature.

## Methods

### Materials

All reagents were used as received without further purification. Chlorobenzene was purchased from Fisher Scientific. 2% Hellmanex III detergent, 1-chloronaphthalene (1-CN), acetone, isopropanol, zinc acetate dehydrate, ethanolamine, and 2-methoxyethanol were purchased from Sigma-Aldrich. PBDB-T-2F (PM6) (*M*_n_: 60,551 g/mol, *D̵*: 2.09) and Y6 (BTP-4F) were purchased from Ossila. MoO_3–*x*_ and silver for thermal evaporation were purchased from the Kurt J. Lesker Company. ITO-patterned glass substrates (*R*_s_ = 15 Ω/sq) were acquired from Xin Yan Technology Limited.

### Flash Differential Scanning Calorimetry

A Mettler-Toledo flash differential scanning calorimeter (Flash DSC 2+) was used. It was equipped with an ultrafast standard chip with heating and cooling rates up to 4000 K/s. All measurements were done under nitrogen gas with a flow rate of 60 mL/min at ambient pressure.

### Wide Angle X-ray Scattering

A laboratory beamline system (Xenocs Inc. Xeuss 2.0) with an X-ray wavelength of 1.54 Å and a sample to detector distance of 15 cm was used here. The bulk samples were filled into a capillary tube with a diameter of 1 mm. Scattering images were recorded on a Pilatus 1 M detector (Dectris Inc.) with an exposure time of 15 min for the transmission mode and 2 h for the grazing incidence mode. All measurements were done under vacuum to minimize air scattering. Temperature-dependent scattering measurements were performed in thin-wall capillary tubes. The sample temperature was controlled by an HFSX350 Linkam stage. For GIWAXS, polymeric thin films on a silicon substrate were annealed ex situ and then measured with an incidence angle of 0.2°. Data were processed using the Irena and Nika package and WAXSTools package in Igor Wavemetrics software.

### Atomic Force Microscopy–Infrared Spectroscopy

Spin-cast films were measured using a nanoIR3 AFM–IR from Bruker Instruments (Santa Barbara, CA) coupled to a MIRcat-QT quantum cascade, mid-infrared laser (frequency range of 917–1800 and 1900–2230 cm^–1^ using a range of pulse frequencies between 355 and 1382 kHz). AFM–IR data were collected in the tapping mode by using a gold-coated AFM probe (spring constant of 40 N/m and resonant frequency of 300 kHz) sourced from Bruker. The pulsed mid-IR laser was tuned to frequencies unique to each component, as determined by FTIR characterization. Acquired images were flattened by using Analysis Studio software.

### Optical Microscopy

The thin films and solar cells were examined under a Zeiss Axio Imager A2m Polarizing Microscope equipped with an Axiocam 305 color camera. The images were collected at 50× magnifications.

### Fabrication of Solar Cells

#### Substrate Preparation

ITO-patterned glass substrates (*R*_s_ = 15 Ω/sq) were cleaned by successive sonication for 20 min in 2% Hellmanex III detergent in Millipore water, acetone, and isopropyl alcohol and then stored under isopropyl alcohol until use. All substrates were blown dry with nitrogen and UV/plasma cleaned for 20 min immediately prior to use.

#### ZnO Layer

ZnO sol–gel precursor solutions were prepared by dissolving zinc acetate dihydrate (108 mg, 0.49 mmol) and ethanolamine (30 μL, 0.50 mmol) in 2-methoxyethanol (1.0 mL) and stirring vigorously overnight. The solution was then filtered through a 0.45 μm PTFE syringe filter; 70 μL of solution was spin-coated onto ITO-coated glass at 5000 rpm for 60 s with an acceleration of 2500 rpm/s. The films were then annealed at 180 °C for 15 min and placed in a nitrogen-filled glovebox. The thickness of the ZnO layer was approximately 30 nm.

#### PM6/Y6 Layer

PM6/Y6 (1:1.2 (w/w), 15.4 mg/mL total concentration) solutions in chlorobenzene were prepared inside a nitrogen-filled glovebox. 0.5% 1-CN (v/v) was added as an additive. The solutions were stirred overnight at 80 °C. The solutions were then filtered through a 1 μm PTFE syringe filter and spin-coated (2 step: 500 rpm for 3 s and 1300 rpm for 25 s) onto glass/ZnO substrates. The films were then stored for 2–4 min in a Petri dish flask and annealed at 100 °C for 10 min. The final thickness of the PM6/Y6 layer was ∼90 nm. Finally, MoO_3–*x*_ (7 nm with a deposition rate of 0.1 Å/s) and Ag (80 nm with a deposition rate of 0.2 Å/s to 15 nm and then 0.5 Å/s for the rest of 65 nm) were thermally evaporated at a base pressure of 3 × 10^–6^ mbar.

#### Solar Cells Aging Test

The thermal stability analyses of solar cells were performed inside a nitrogen-filled glovebox on temperature-controlled hot plates. To study the effect of thermal aging on the PM6/Y6 layer, different aging times and temperatures were used. All devices were measured and stored inside a nitrogen-filled glovebox. *J*–*V* characteristics of aged OSC devices were measured at different times using a standardized setup made of AM1.5G illumination of 100 mW/cm^2^ using an AAB class solar simulator from ABET Technologies with a Xenon Arc lamp. The illumination was calibrated to 1 sun by using a SSIVT-REF (125-9007) reference silicon solar cell.

## Supporting Information Available

Glass transition temperatures measurements for Y6 (flash DSC) and PM6 (DMA), enthalpy changes upon crystallization for Y6 and PM6/Y6 blend, isothermal crystallization kinetics for Y6 and PM6/Y6 blend, WAXS and GIWAXS 1D/2D information for Y6 and PM6/Y6 blend, AFM IR and calibration curve establishment for the PM6/Y6 BHJ layer, device performance data for the PM6/Y6 blend under various thermal conditions, and AFM IR for actual solar cell devices and tables for isothermal crystallization kinetics fitting parameters, 1:1 PM6/Y6 GIWAXS scattering peak fitting results, and device performance data (PDF)
