# Supplementary material for: Effect of Thermal Stress on Morphology in High-Performance Organic Photovoltaic Blends
Source: JACS Au. 2024 Oct 10;4(11):4334–44. doi: 10.1021/jacsau.4c00631 (PMC11600174; doi:10.1021/jacsau.4c00631)
Supplement: Supplementary file 1 — au4c00631_si_001.pdf [file au4c00631_si_001.pdf]

The Effect of Thermal Stress on the Morphology in High-Performance Organic Photovoltaic Blends

*Haoyu Zhao[#], Nathaniel Prine[#], Soumya Kundu, Guorong Ma, Xiaodan Gu[1]**

School of Polymer Science and Engineering, Center for Optoelectronic Materials and Devices, The University of Southern Mississippi, Hattiesburg, MS 39406, USA.

[#] These authors contributed equally to this work.

This supplemental information includes

Figure S1 to S24

glass transition temperatures measurements for Y6 (Flash DSC) and PM6 (DMA), enthalpy changes upon crystallization for Y6 and PM6:Y6 blend, isothermal crystallization kinetics for Y6 and PM6:Y6 blend, WAXS and GIWAXS 1D/2D information for Y6 and PM6:Y6 blend, AFM IR and calibration curve establishment for PM6:Y6 BHJ layer, device performance data for PM6:Y6 blend under various thermal conditions, AFM IR for actual solar cell devices

Table S1 to S5

isothermal crystallization kinetics fitting parameters, 1:1 PM6:Y6 GIWAXS scattering peak fitting results, Device performance data

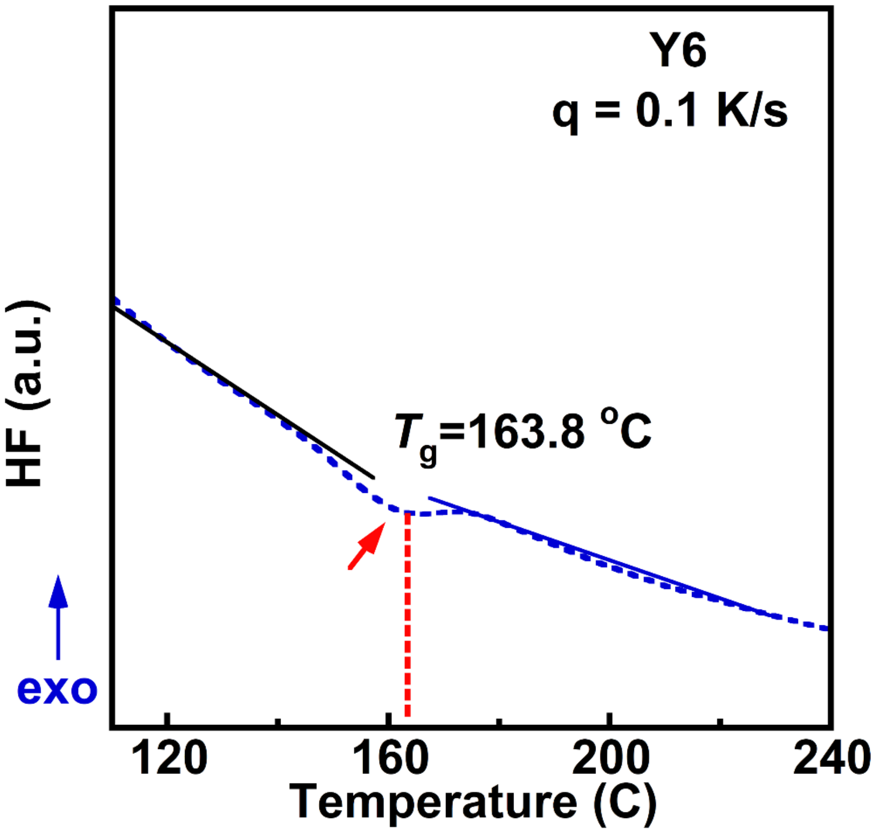

Figure S1. Flash DSC of neat Y6, where the cooling rate is 0.1 K/s and the arrow indicates the glass transition temperature

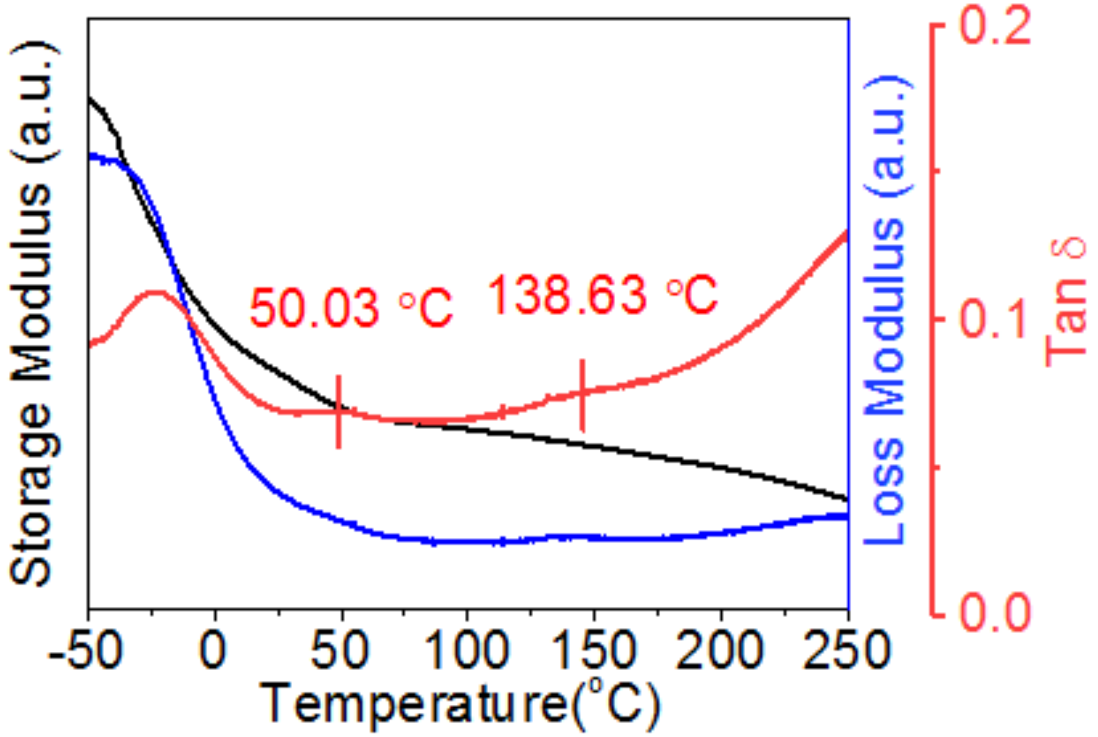

Figure S2. DMA characterization for PM6 sample. The results shows two glass transition temperatures related to polymer backbone as indicated by a vertical dash.

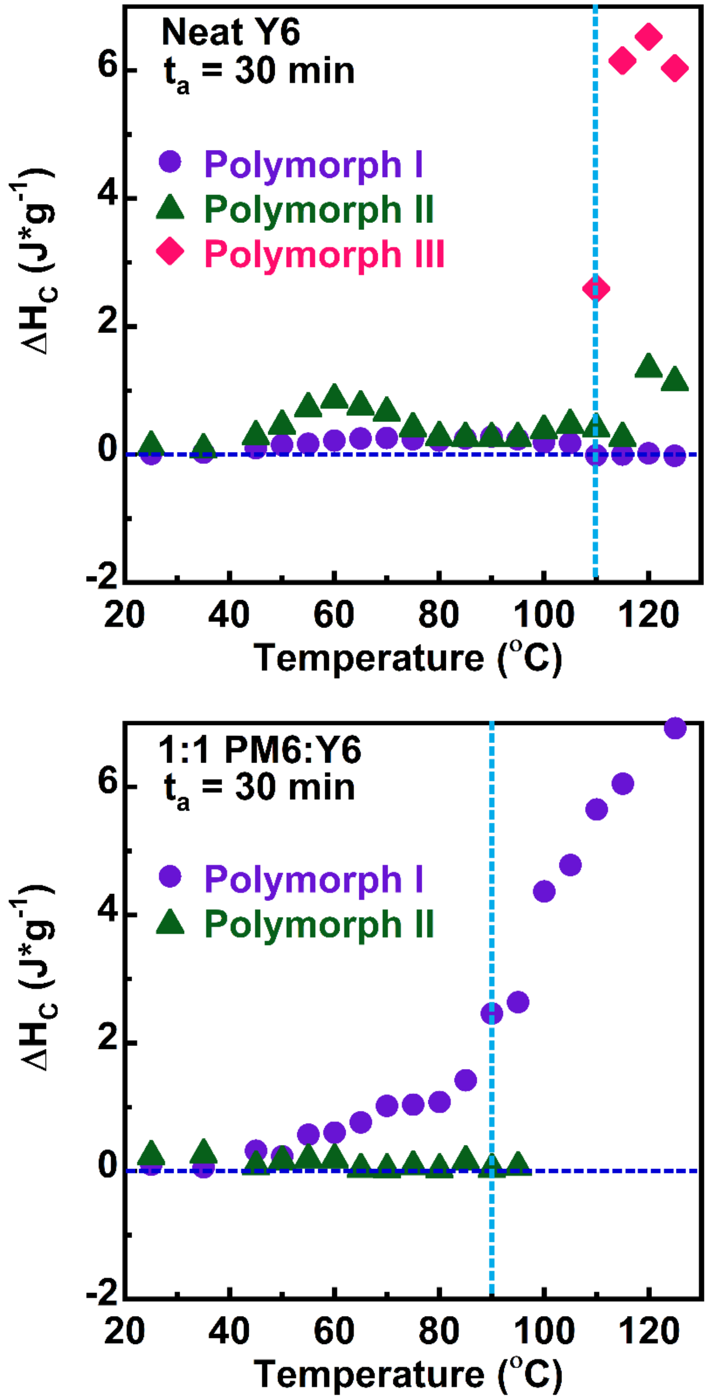

Figure S3. Enthalpy changes (ΔH_C) upon crystallization against annealing temperature for neat Y6 (top panel) and PM6:Y6 (bottom panel), where polymorphs are named orderly as the crystallization peak temperatures increase from R.T. to the end of heating scan.

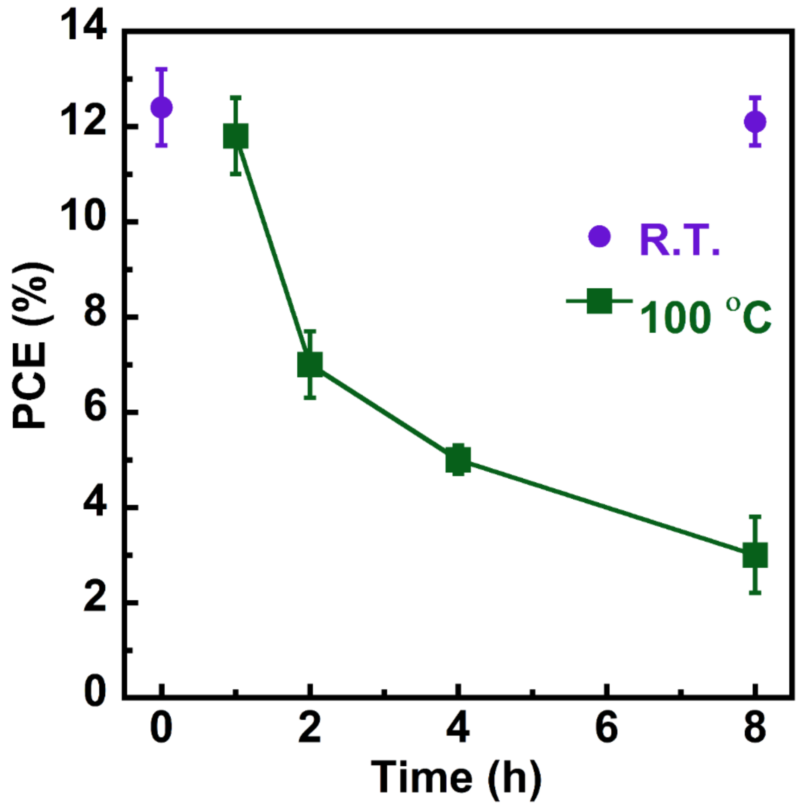

Figure S4. PCE against operational time for half of the device that is isothermally annealed devices. Here the half device refer to the device with ITO/ZnO/BHJ, but without the MoO_3 and Ag electrode.

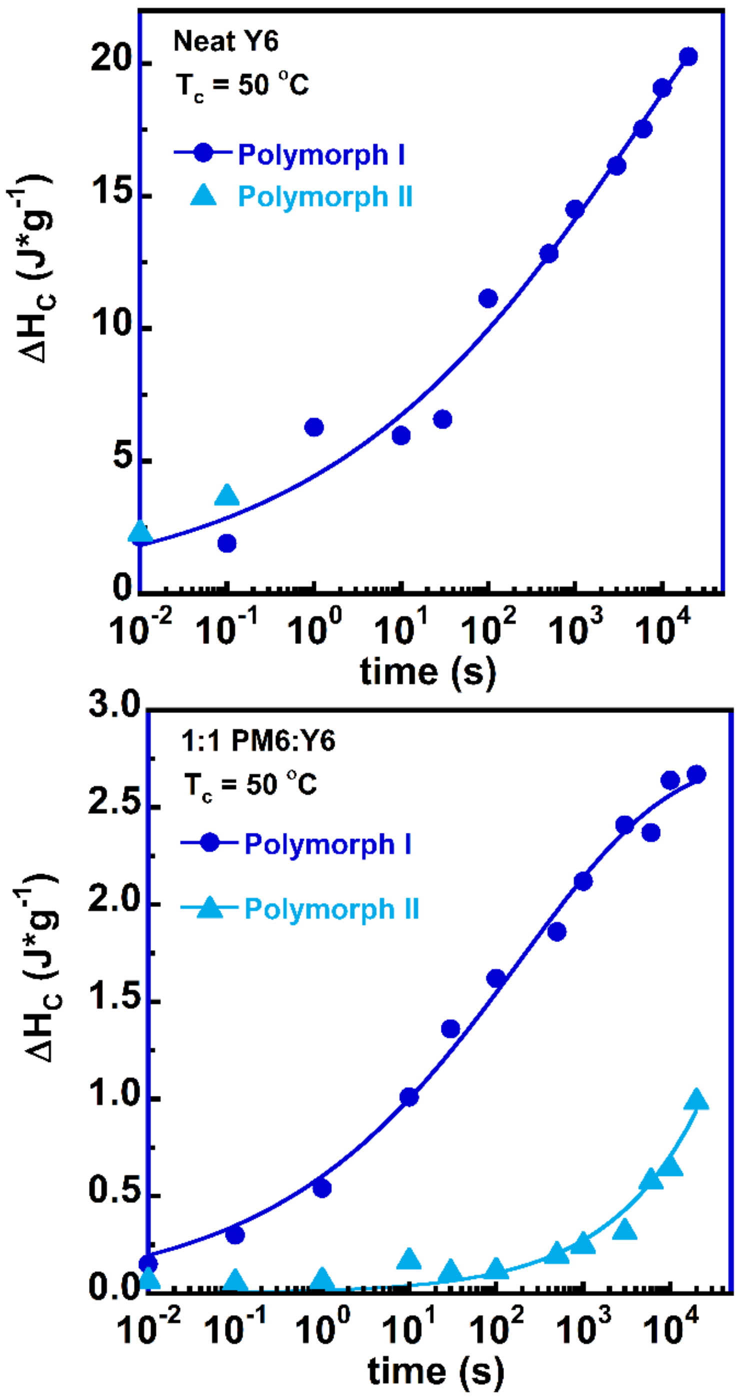

Figure S5. Enthalpy changes upon crystallization against isothermal annealing times at 50 °C for neat Y6 (top panel) and PM6:Y6 (bottom panel)

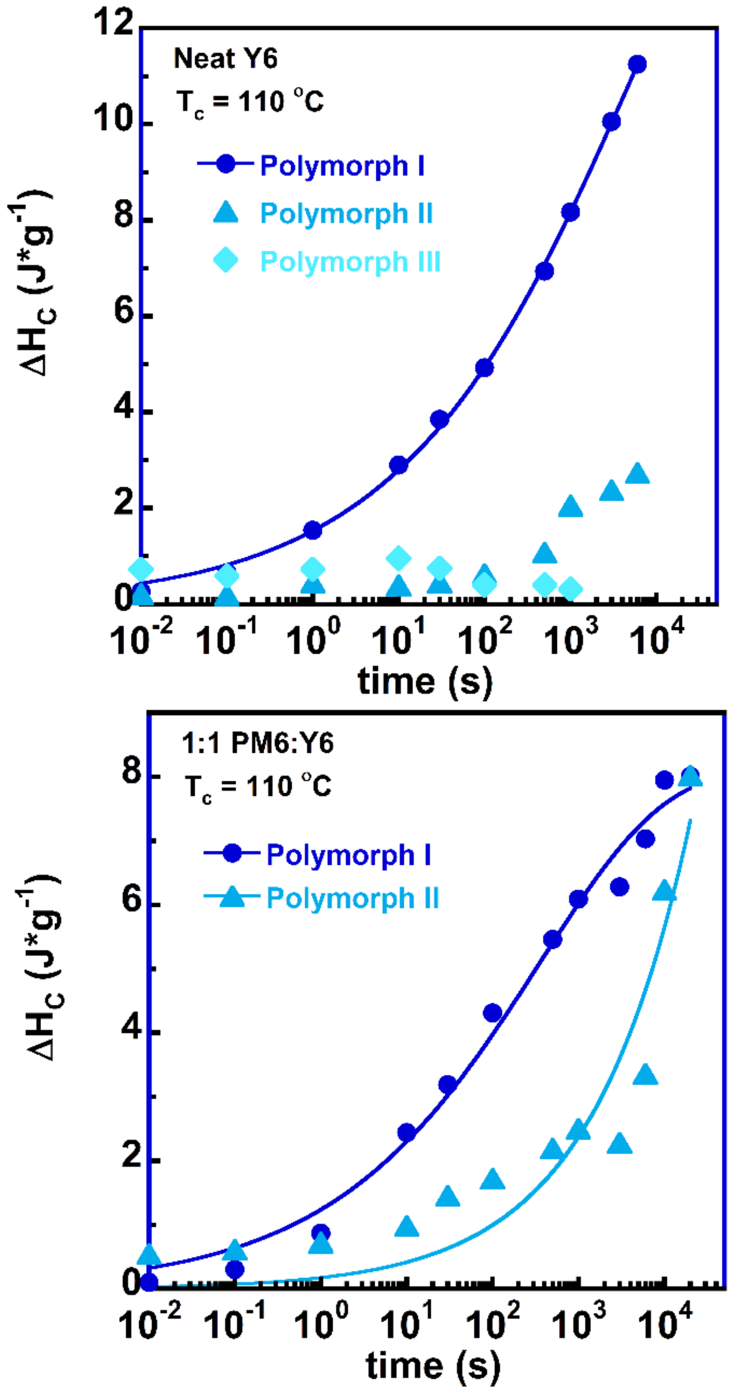

Figure S6. Enthalpy changes upon crystallization against isothermal annealing times at 110 °C for neat Y6 (top panel) and PM6:Y6 (bottom panel)

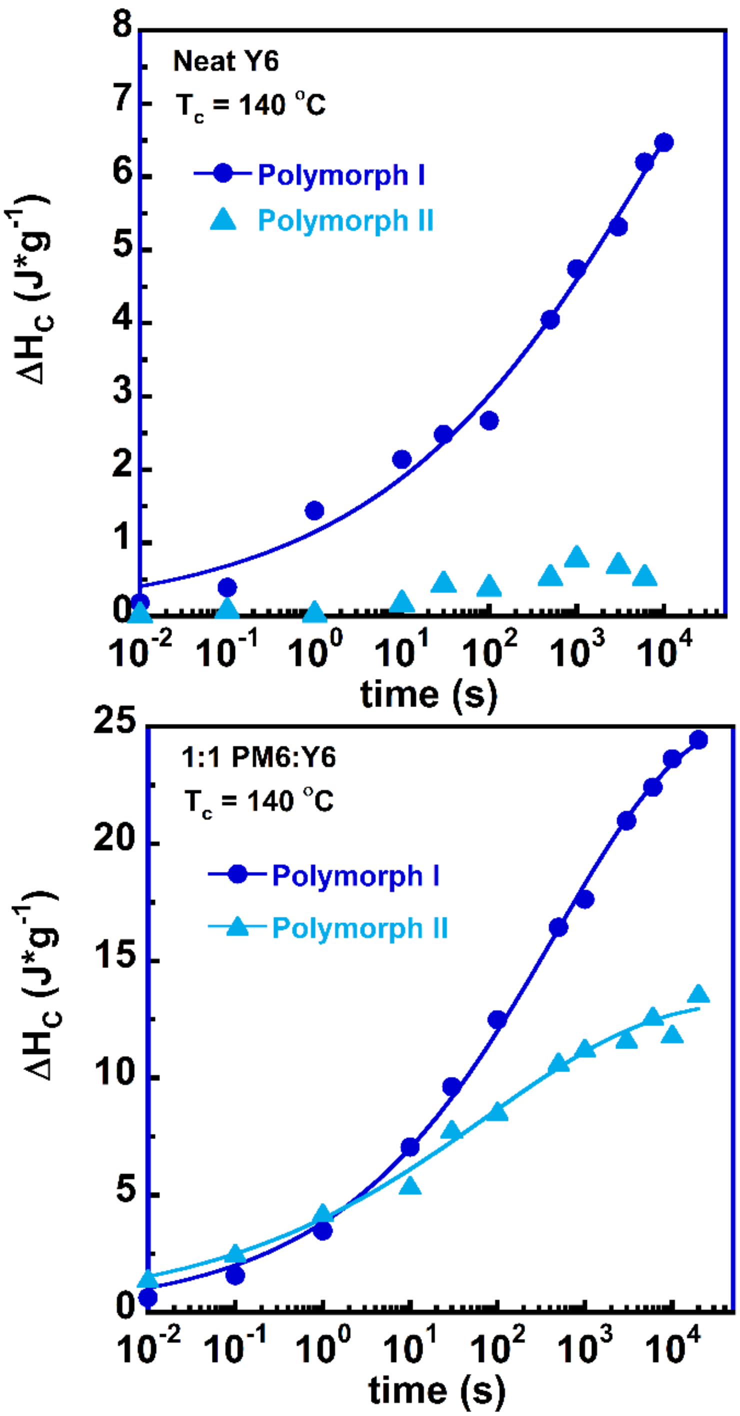

Figure S7. Enthalpy changes upon crystallization against isothermal annealing times at 140 °C for neat Y6 (top panel) and PM6:Y6 (bottom panel)

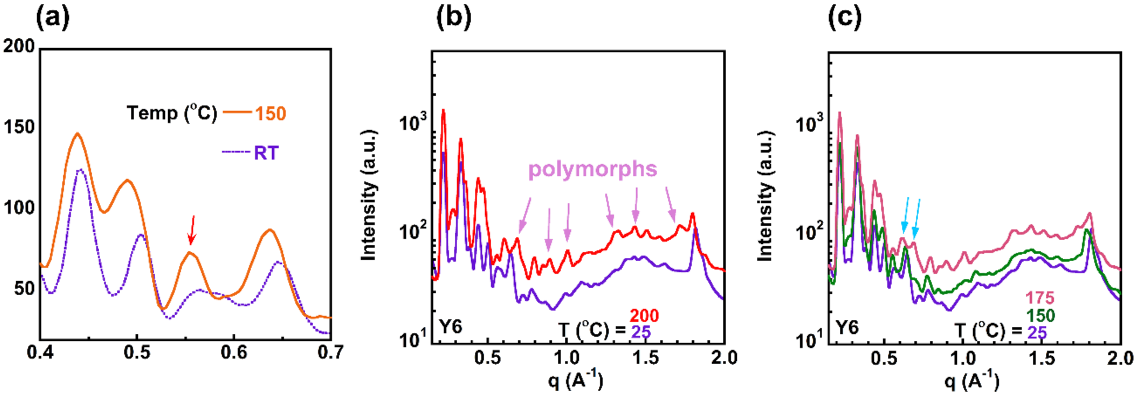

Figure S8 neat Y6 WAXS data: **a).**polymorph I formed at Y6 heated to 150 °C **b).** polymorph II formed at Y6 heated to 200 °C **c).** scattering peak separated into two peaks as temperature increased from 150 to 175 °C for neat Y6

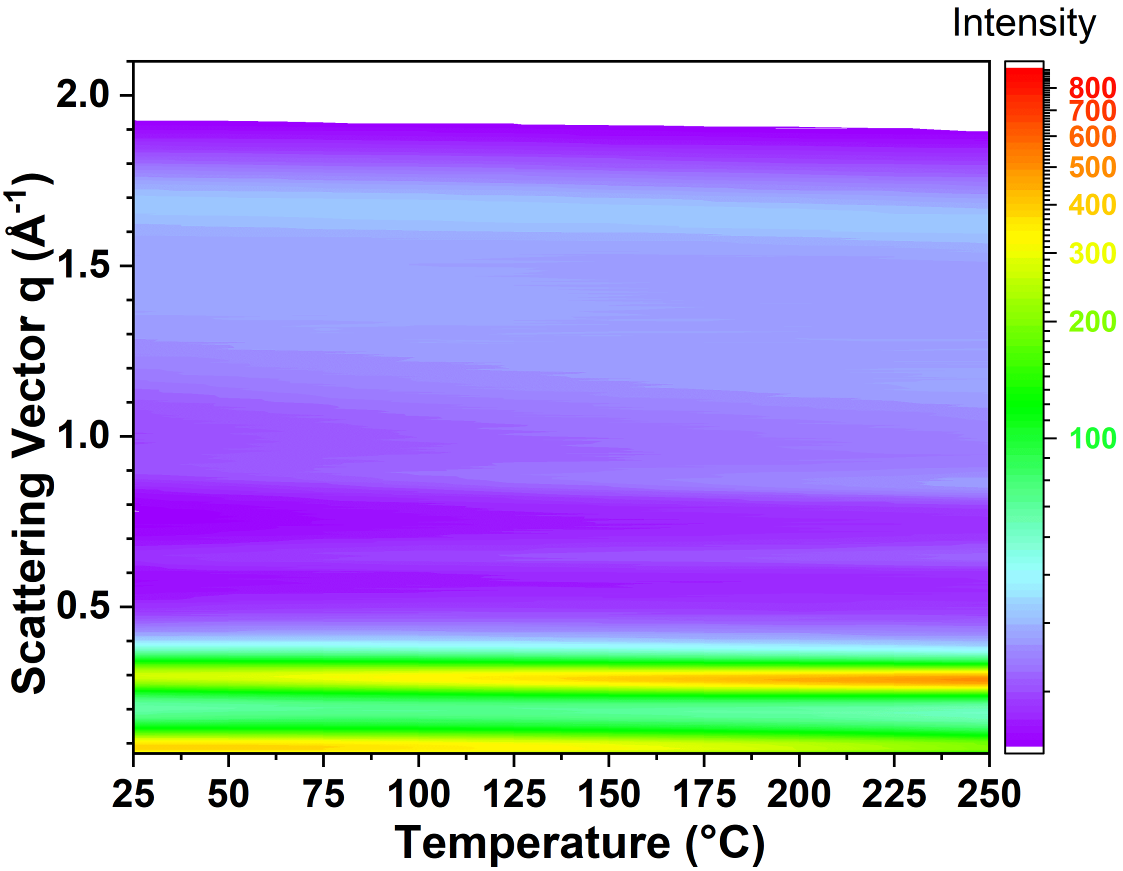

Figure S9. Intensity map for *in-situ* heating PM6 WAXS experiments

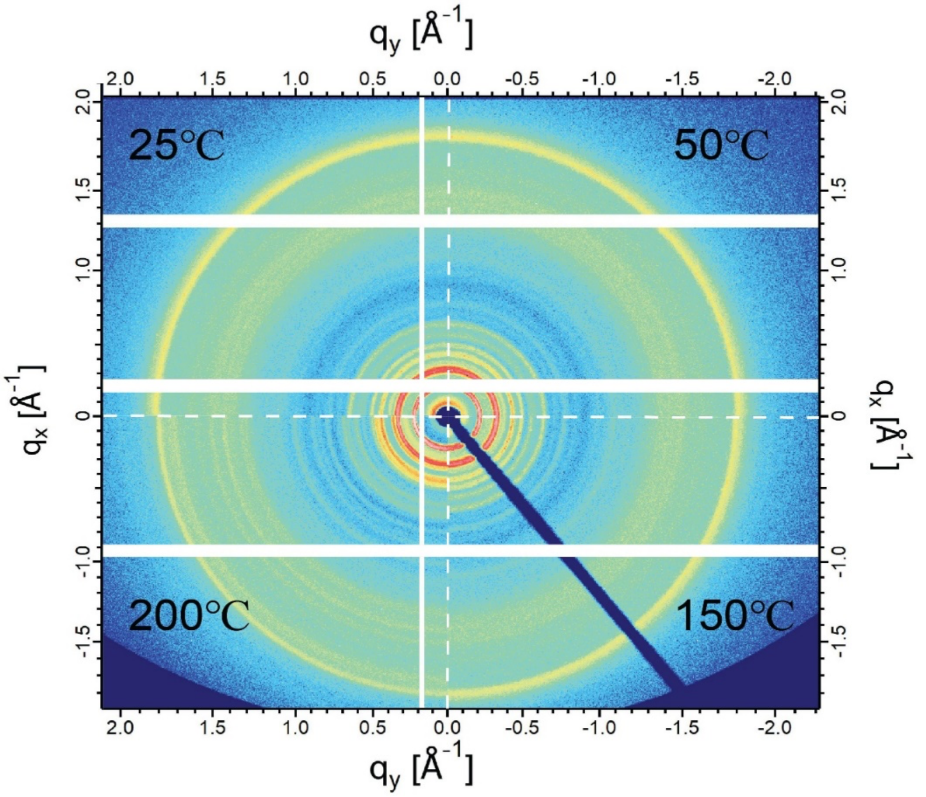

Figure S10. Neat Y6 *insitu* heating WAXS 2D images four different quadrants represent four different annealing temperature (25, 50, 150 and 200 ^{0}C) for comparison.

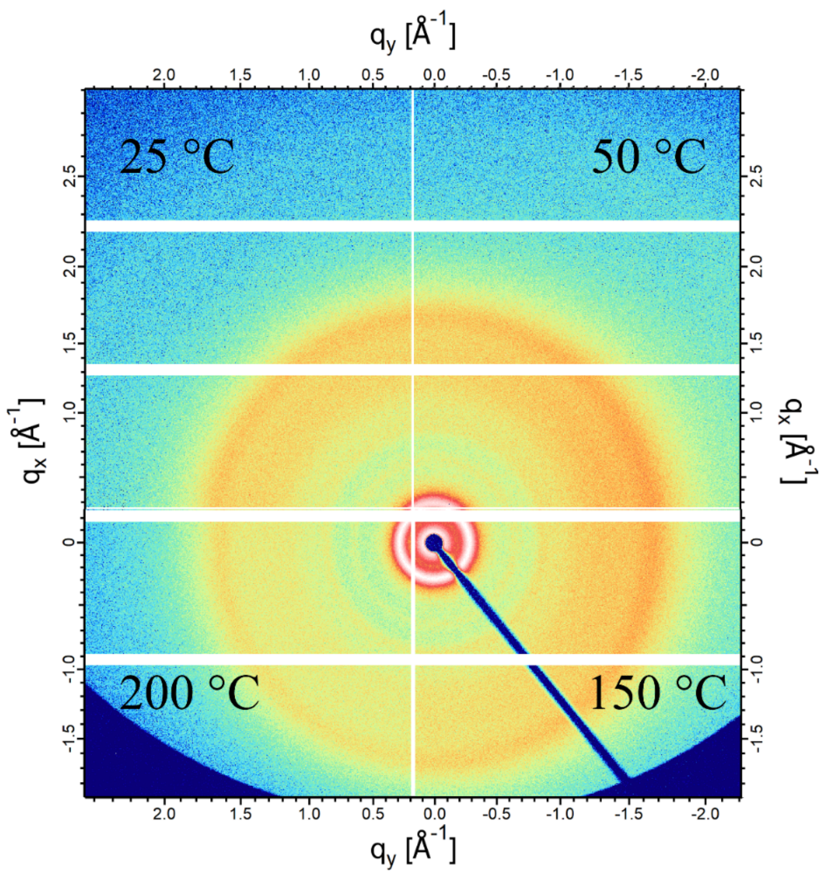

Figure S11. Neat PM6 *insitu* heating WAXS 2D images four different quadrants represent four different annealing temperature (25, 50, 150 and 200 ^{0}C) for comparison.

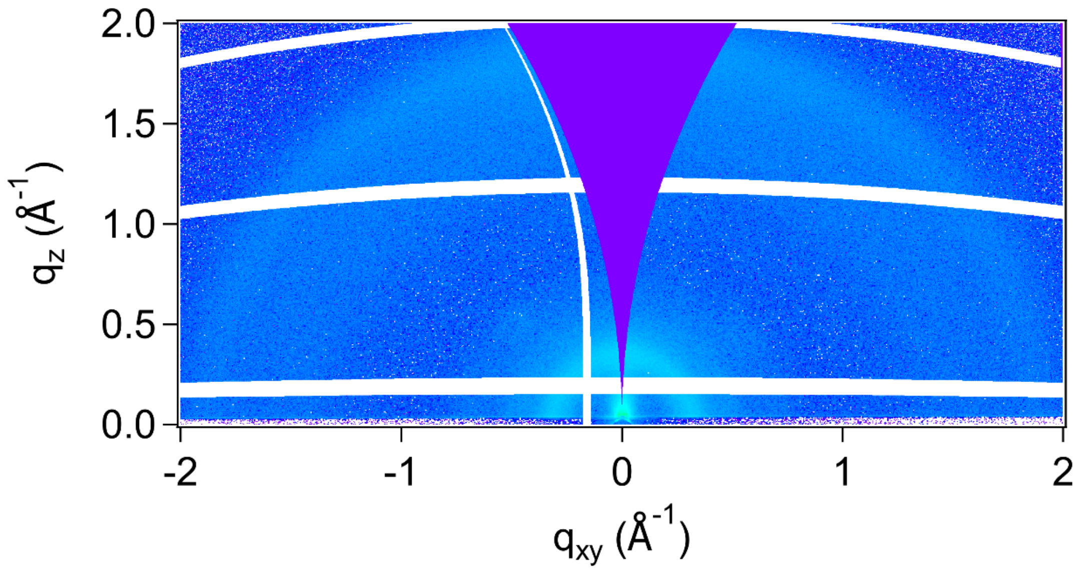

Figure S12. PM6:Y6 spincoated film on wafer *exsitu* heating at R.T. GIWAXS 2D images

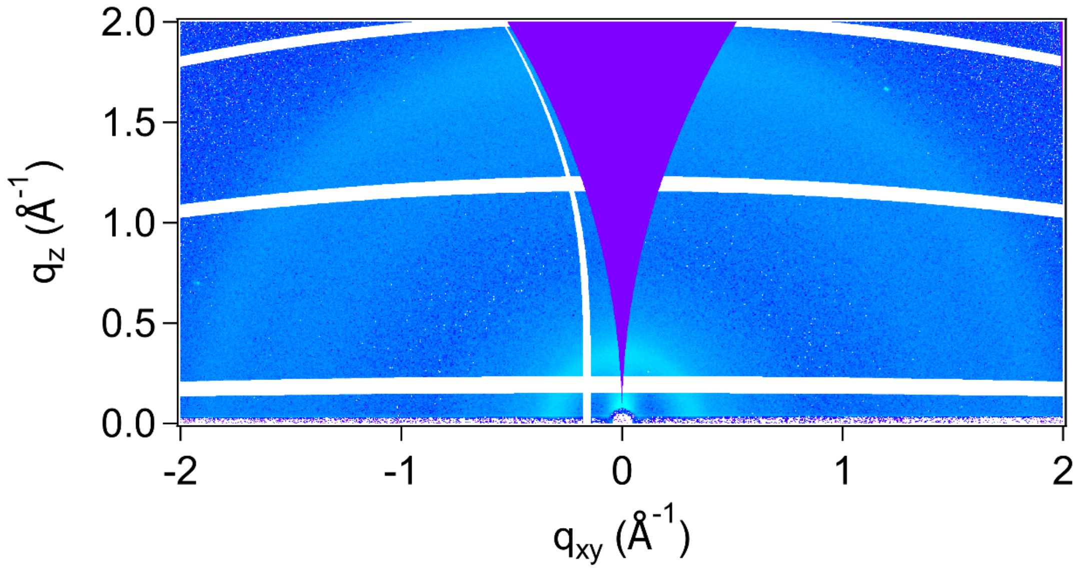

Figure S13. PM6:Y6 spincoated film on wafer *exsitu* heating at 50 °C GIWAXS 2D images

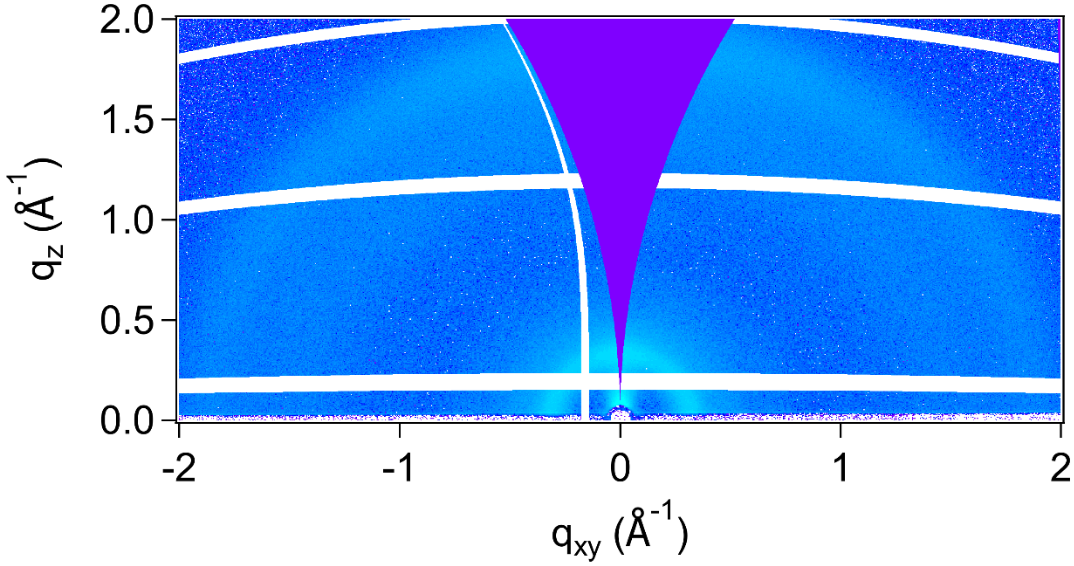

Figure S14. PM6:Y6 spincoated film on wafer *exsitu* heating at 100 °C GIWAXS 2D images

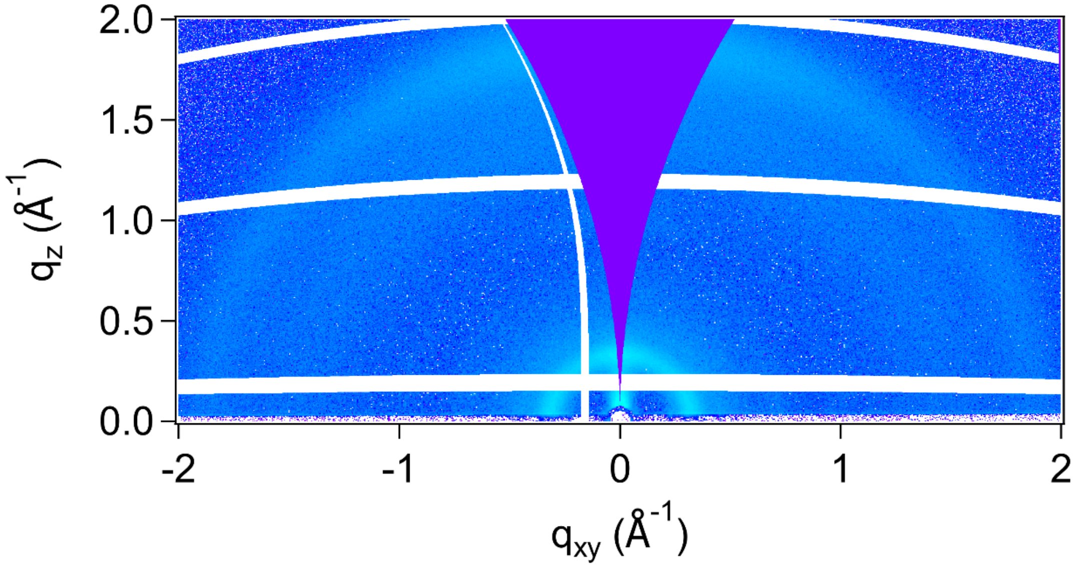

Figure S15. PM6:Y6 spincoated film on wafer *exsitu* heating at 150 °C GIWAXS 2D images

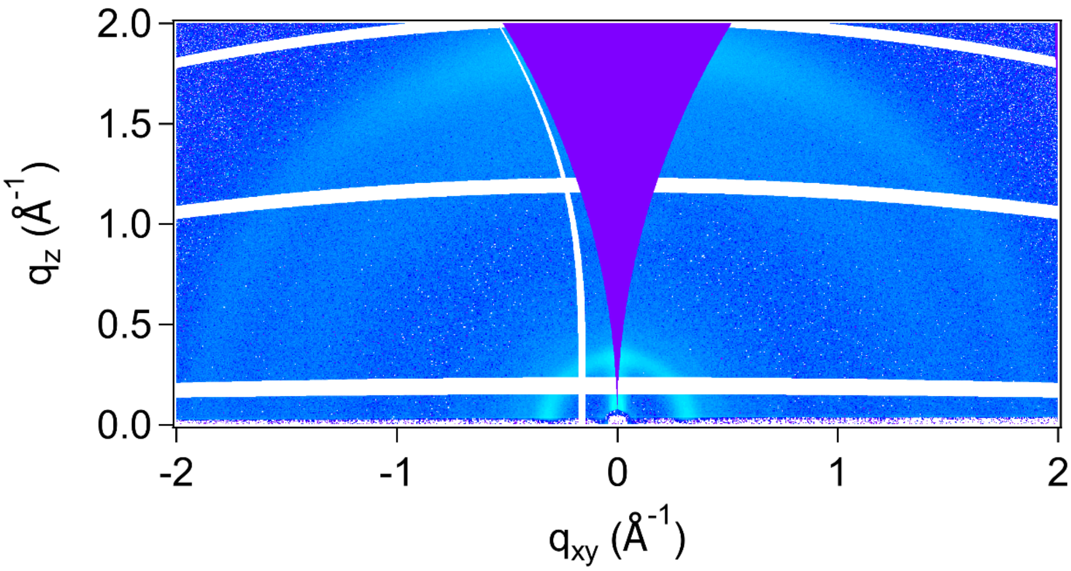

Figure S16. PM6:Y6 spincoated film on wafer *exsitu* heating at 200 °C GIWAXS 2D images

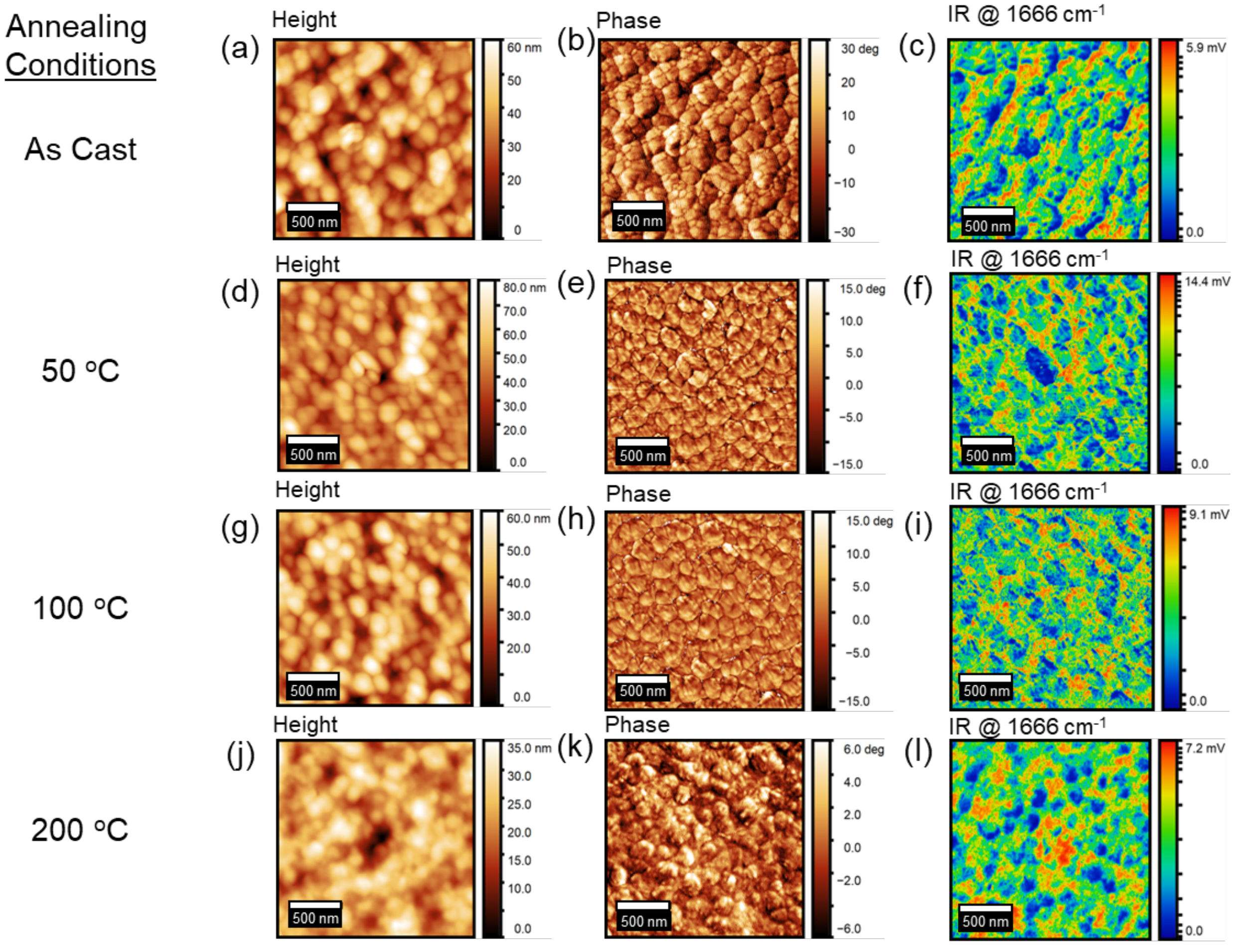

Figure S17. Height (a,d,g,j), phase (b,e,h,k), and IR images (c,f,i,l) of PM6:Y6 blends annealed at RT, 50 °C, 100 °C, and 200 °C.

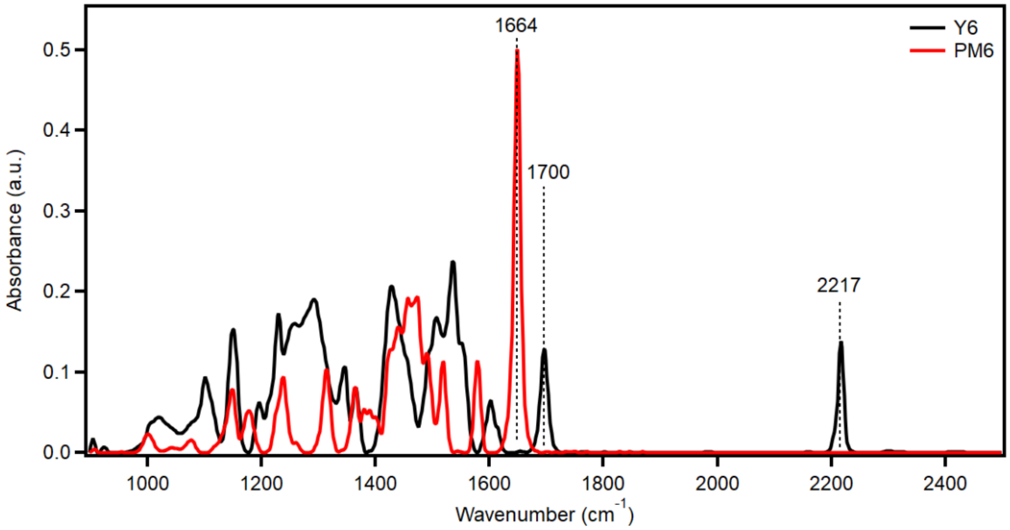

Figure S18. Bulk FTIR spectra for neat PM6 and neat Y6.

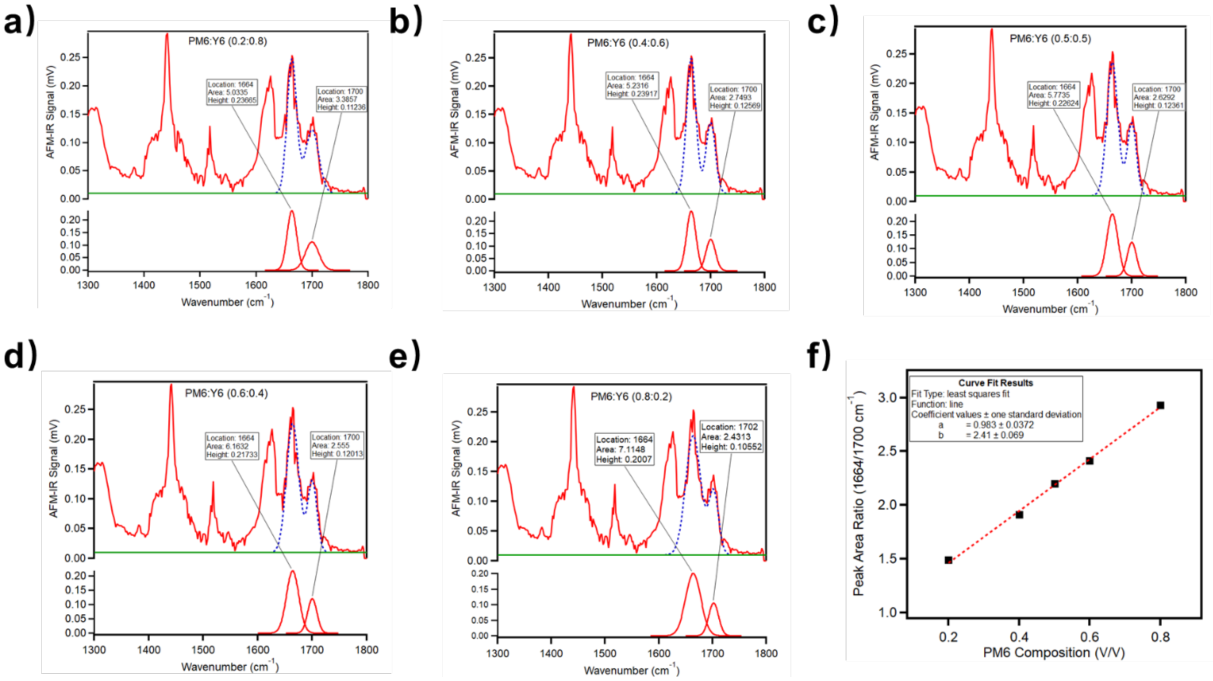

Figure S19. AFM-IR spectra and fit curves for PM6:Y6 blends prepared to ratios of **a)** 0.2:0.8, **b)** 0.4:0.6, **c)** 0.5:0.5, **d)** 0.6:0.4, and **e)** 0.8:0.2 PM6:Y6. **f)** Calibration curve calculated using the composition as the X-values and the area under the unique peak for each component as the Y-component.

A note on the calibration curve construction:

To further investigate the domain composition and purity of the localized morphology, we constructed a calibration curve using PM6:Y6 films prepared with incrementally increasing PM6 concentration ratios (PM6:Y6 ratios of 20/80, 40/60, 50/50, 60/40, and 80/20). We measured the AFM-IR response for these films, while ensuring consistent thickness *via* AFM measurements. From the bulk FTIR plot of **Figure S18**, we can observe unique absorption peaks for PM6 at 1666 cm^{-1} and for Y6 at 1702/2217 cm^{-1}. Since an unbiased baseline is needed to establish the calibration curve, we then plotted the ratio of the PM6 peak area at 1666 cm^{-1} and the Y6 peak area at 1702 cm^{-1} as a function of Y6 concentration (**Figure S19**). This linear curve served as a reference for calculating the location-dependent chemical composition of annealed PM6:Y6 blends. A detailed discussion of this quantitative approach is listed in our previous work.

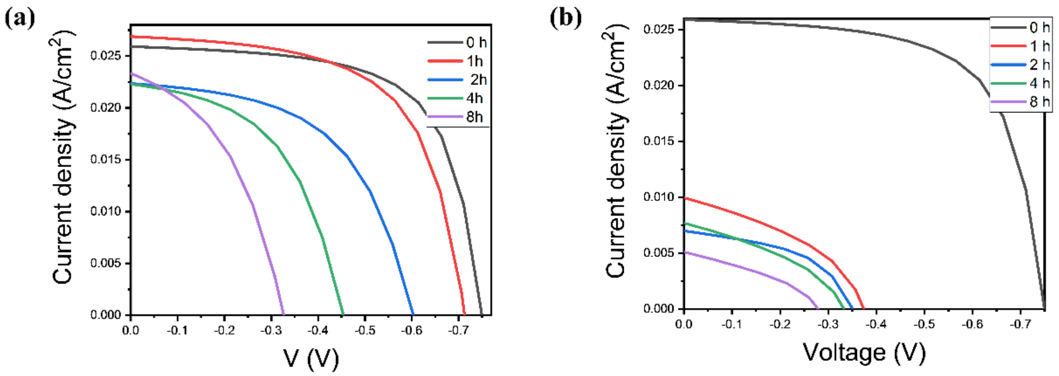

Figure S20. Representative *J-V* curves of ITO/ZnO/PM6:Y6/MoO_3/Ag OPVs thermally aged at (a) 100 °C (b) 200 °C with different aging time (0h, 1h, 2h, 4h, and 8h).

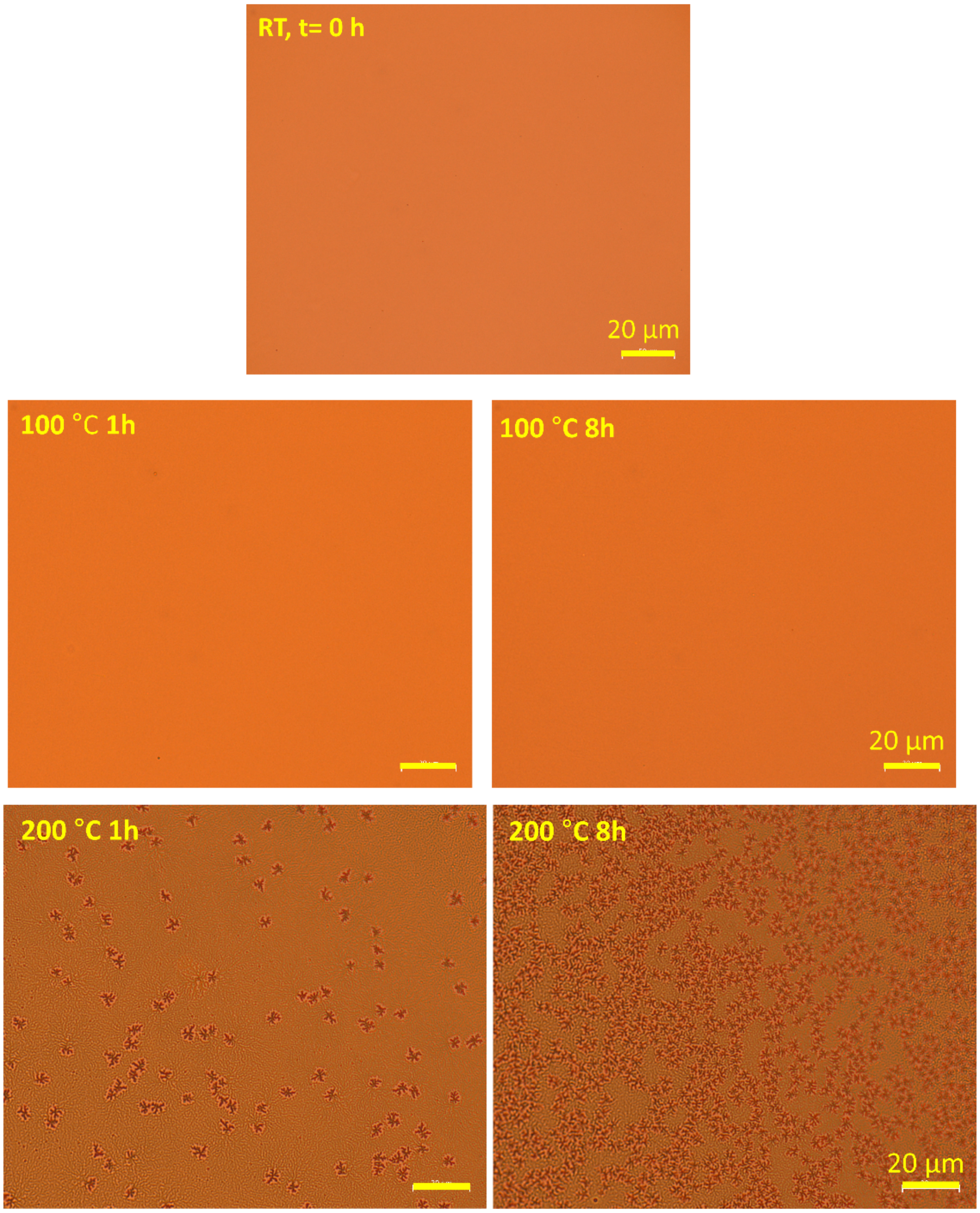

Figure S21. top panel: Optical microscopy image of 1:1.2 PM6:Y6 blend layer at room temperature at 50x resolution (control). **mid panel:** Optical microscopy image of 1:1.2 PM6:Y6 blend layer after annealing at 100 °C for 1h and 8 h. Images are taken at 50x resolution. **Bottom panel:** Optical microscopy image of 1:1.2 PM6:Y6 blend layer after annealing at 200 °C for 1h and 8 h. Images are taken at 50x resolution.

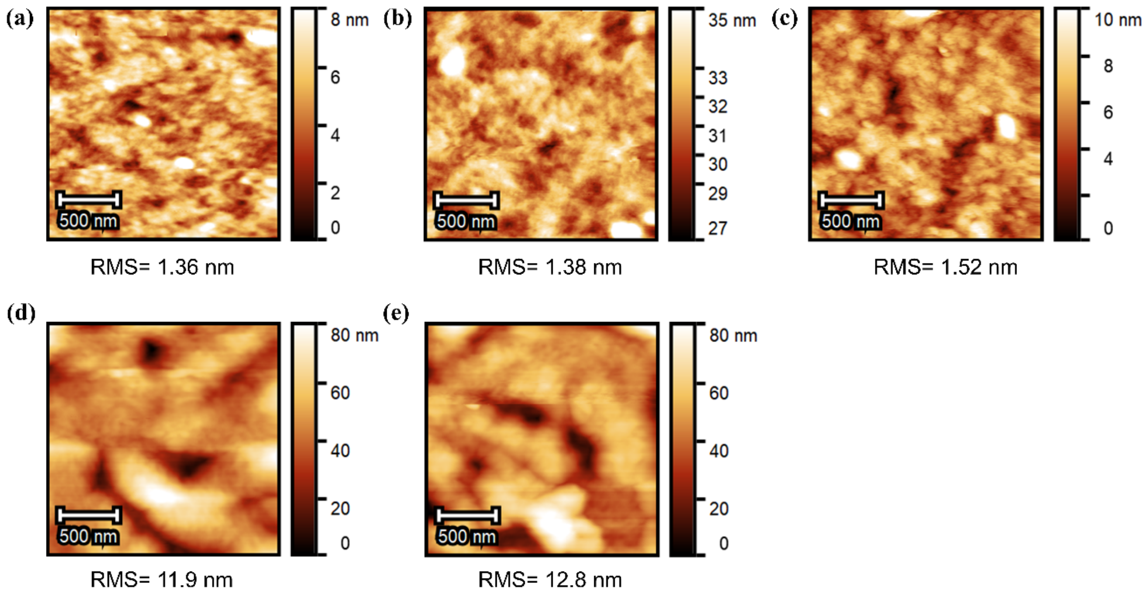

Figure S22. AFM height images for **a)** R.T. aged 8h, **b)** 100 °C annealed 1h, **c)** 100 °C annealed 8h, **d)** 200 °C annealed 1h, **e)** 200 °C annealed 8h

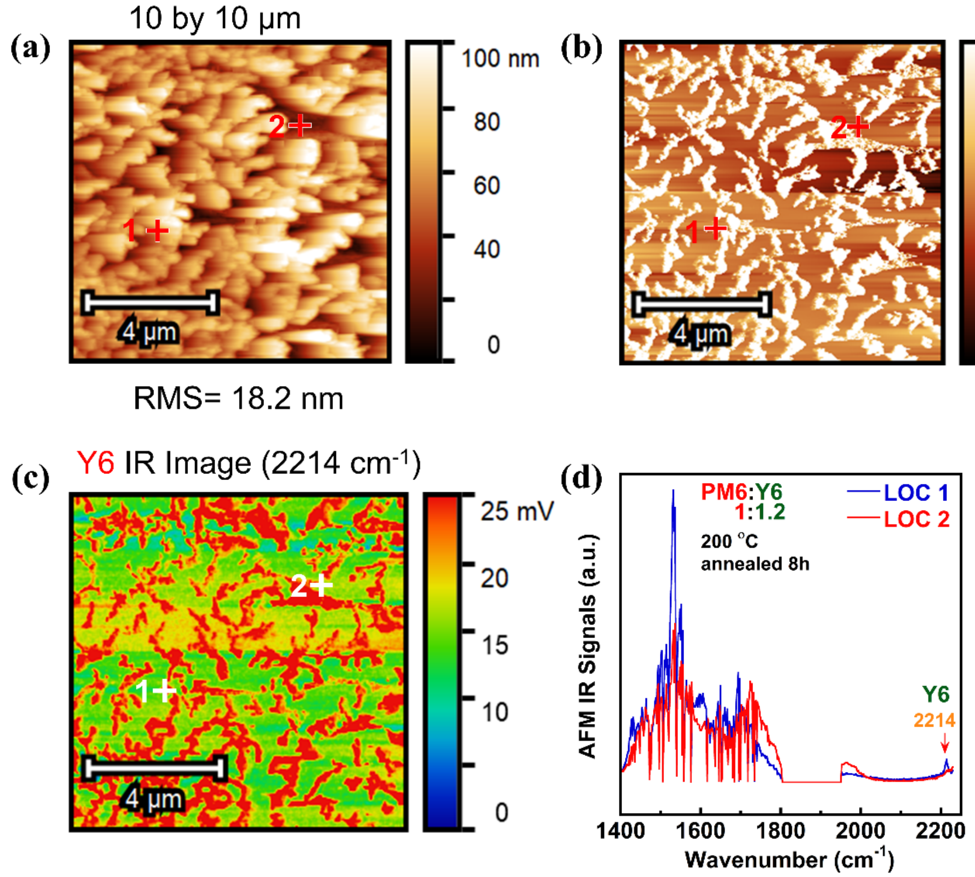

Figure S23. AFM-IR scanned on 200 °C annealed 8h device **a)** 10 by 10 μm height image, **b)** phase image, **c)** scan at Y6 absorption peak at 2214 cm^{-1}, **d)** IR Spectra

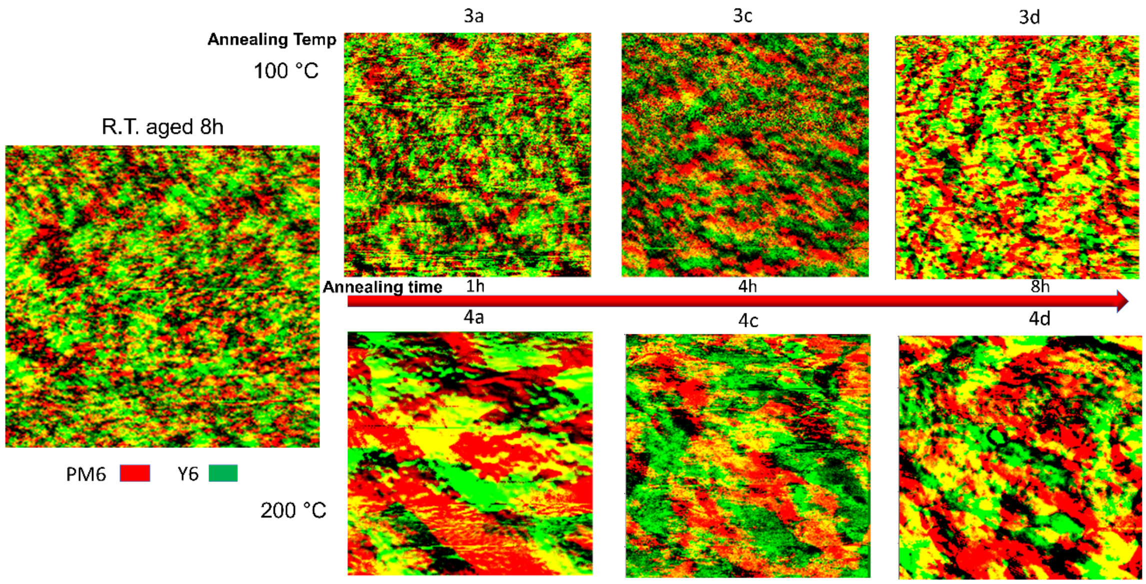

Figure S24. AFM-IR overlay images indicating the annealing temperature/time influences on film morphology, where red area represented PM6 rich domains and green area represented Y6 rich domains

Table S1. Avrami fitting parameters for isothermal crystallization kinetics of neat Y6

Temperature (°C)	k	n	τ (s)
**50**	0.17 ± 0.05	0.20	891.36 ± 125.85
**80**	0.11 ± 0.03	0.27	820.09 ± 183.87
**110**	0.09 ± 0.01	0.28	1201.9 ± 12.0
**140**	0.12 ± 0.03	0.23	1542.0 ± 179.3

Table S2. Avrami fitting parameters for isothermal crystallization kinetics of PM6:Y6

Temperature (°C)	k	n	τ (s)
**50**	0.25 ± 0.02	0.26	48.83 ± 0.81
**70**	0.25 ± 0.02	0.27	44.90 ± 0.86
**80**	0.34 ± 0.04	0.20	33.42 ± 1.25
**90**	0.22 ± 0.02	0.26	82.62 ± 1.53
**110**	0.17 ± 0.03	0.30	108.73 ± 4.42
**140**	0.16 ± 0.01	0.3	133.75 ± 0.74

Table S3. 1:1 PM6:Y6 GIWAXS 1d fitting results

Temperature (°C)	q (Å^{-1})	FHWM	q (Å^{-1})	FHWM
**25**	0.67	0.21	N/A	N/A
**50**	0.66	0.22	N/A	N/A
**100**	0.69	0.17	N/A	N/A
**150**	0.69	0.16	0.99	0.15
**200**	0.70	0.11	0.98	0.09

Table S4. OPV performance optimization for R.T. as cast active lay

# of devices	Annealing (°C)	Time (hrs)	PCE (%)	FF (%)	Jsc (mA cm^{-2})	Voc (V)
**17**	RT	0 h	12.4 ± 0.8	62.2 ± 1.5	26.3 ± 1.4	0.755 ± 0.021
**29**	RT	8 h	12.1 ± 0.5	62.1 ± 1.9	26.3 ± 1.4	0.741 ± 0.0167

Table S5. OPV parameters at different time and annealing conditions

# of devices	Annealing (°C)	Time (days)	PCE (%)	FF (%)	Jsc (mA cm^{-2})	Voc (V)
11	RT	1	9.3 ± 0.4	61.3 ± 2.6	21.9 ± 2.0	0.694 ± 0.031
11	RT	2	9.4 ± 0.4	61.9 ± 3.0	21.8 ± 2.0	0.703 ± 0.029
11	RT	3	9.5 ± 0.4	62.2 ± 2.9	21.8 ± 2.0	0.704 ± 0.030
11	RT	4	9.5 ± 0.3	62.2 ± 3.2	21.7 ± 2.0	0.706 ± 0.028

11	RT	5	9.4 ± 0.4	61.8 ± 3.8	21.7 ± 2.0	0.705 ± 0.031
11	RT	6	9.3 ± 0.4	62.2 ± 3.6	21.2 ± 2.0	0.710 ± 0.028
11	RT	8	9.2 ± 0.3	62.1 ± 3.4	20.9 ± 2.0	0.709 ± 0.030
10	RT	12	9.2 ± 0.3	62.1 ± 4.1	21.0 ± 2.0	0.711 ± 0.026
10	RT	15	9.2 ± 0.5	62.0 ± 3.4	20.8 ± 2.0	0.713 ± 0.0267
10	100	1	10.1 ± 0.4	63.3 ± 1.1	22.0 ± 0.9	0.724 ± 0.011
10	100	2	9.5 ± 0.3	61.6 ± 1.4	21.8 ± 0.9	0.713 ± 0.010
10	100	3	9.2 ± 0.5	60.6 ± 1.6	21.4 ± 1.0	0.706 ± 0.009
10	100	4	8.9 ± 0.5	60.1 ± 1.6	21.1 ± 1.0	0.703 ± 0.009
10	100	5	8.8 ± 0.5	59.8 ± 1.5	20.8 ± 1.0	0.704 ± 0.011
10	100	6	8.4 ± 0.3	59.2 ± 1.4	20.3 ± 0.8	0.702 ± 0.012
11	100	8	8.0 ± 0.4	57.7 ± 2.0	20.0 ± 0.7	0.695 ± 0.015

11	100	12	7.6 ± 0.5	56.4 ± 2.0	19.6 ± 0.9	0.686 ± 0.017
10	100	15	7.2 ± 0.5	54.8 ± 2.0	19.3 ± 0.9	0.684 ± 0.012
12	150	1	7.7 ± 0.6	54.1 ± 1.6	20.8 ± 1.4	0.683 ± 0.006
12	150	2	7.2 ± 0.5	52.1 ± 1.4	20.2 ± 1.3	0.682 ± 0.005
15	150	3	6.3 ± 0.5	49.1 ± 1.7	19.4 ± 1.4	0.667 ± 0.027
16	150	4	5.9 ± 0.5	47.3 ± 1.8	18.8 ± 1.4	0.663 ± 0.025
13	150	5	5.7 ± 0.4	46.7 ± 2.2	18.7 ± 1.0	0.656 ± 0.027
14	150	6	5.2 ± 0.5	45.1 ± 2.1	17.6 ± 1.4	0.655 ± 0.023
14	150	8	4.7 ± 0.4	43.5 ± 2.0	17.0 ± 1.4	0.642 ± 0.026
13	150	12	4.0 ± 0.4	40.8 ± 2.4	16.0 ± 1.3	0.618 ± 0.026
15	150	15	3.5 ± 0.3	41.0 ± 1.7	16.5 ± 1.5	0.572 ± 0.045
